# The Functions of Mediator in *Candida albicans* Support a Role in Shaping Species-Specific Gene Expression

**DOI:** 10.1371/journal.pgen.1002613

**Published:** 2012-04-05

**Authors:** Nathalie Uwamahoro, Yue Qu, Branka Jelicic, Tricia L. Lo, Cecile Beaurepaire, Farkad Bantun, Tara Quenault, Peter R. Boag, Georg Ramm, Judy Callaghan, Traude H. Beilharz, André Nantel, Anton Y. Peleg, Ana Traven

**Affiliations:** 1 Department of Biochemistry and Molecular Biology, Monash University, Clayton, Victoria, Australia; 2 Department of Microbiology, Monash University, Clayton, Victoria, Australia; 3 Biotechnology Research Institute, National Research Council of Canada, Montreal, Quebec, Canada; 4 Monash Micro Imaging, Monash University, Clayton, Australia; 5 Department of Infectious Diseases, The Alfred Hospital, Melbourne, Victoria, Australia; Wadsworth Center, United States of America

## Abstract

The Mediator complex is an essential co-regulator of RNA polymerase II that is conserved throughout eukaryotes. Here we present the first study of Mediator in the pathogenic fungus *Candida albicans*. We focused on the Middle domain subunit Med31, the Head domain subunit Med20, and Srb9/Med13 from the Kinase domain. The *C. albicans* Mediator shares some roles with model yeasts *Saccharomyces cerevisiae* and *Schizosaccharomyces pombe*, such as functions in the response to certain stresses and the role of Med31 in the expression of genes regulated by the activator Ace2. The *C. albicans* Mediator also has additional roles in the transcription of genes associated with virulence, for example genes related to morphogenesis and gene families enriched in pathogens, such as the *ALS* adhesins. Consistently, Med31, Med20, and Srb9/Med13 contribute to key virulence attributes of *C. albicans*, filamentation, and biofilm formation; and *ALS1* is a biologically relevant target of Med31 for development of biofilms. Furthermore, Med31 affects virulence of *C. albicans* in the worm infection model. We present evidence that the roles of Med31 and Srb9/Med13 in the expression of the genes encoding cell wall adhesins are different between *S. cerevisiae* and *C. albicans*: they are repressors of the *FLO* genes in *S. cerevisiae* and are activators of the *ALS* genes in *C. albicans*. This suggests that Mediator subunits regulate adhesion in a distinct manner between these two distantly related fungal species.

## Author Summary

In this study, we compared the roles of Mediator, a central transcriptional regulator in all eukaryotes, between the pathogenic fungus *Candida albicans* and the non-pathogenic model yeasts *Saccharomyces cerevisiae* and *Schizosaccharomyces pombe*. We discovered that Mediator has both shared and species-specific functions in the three yeasts. The shared functions include regulation of genes required for cell separation after cell division by the Middle domain subunit Med31. The species-specific functions include transcriptional regulation of the cell wall adhesins, which play key roles in the pathogenesis of *C. albicans*. In *C. albicans*, the Mediator subunits Med31, Med20, and Srb9/Med13 are activators of the *ALS* cell wall adhesins. In *S. cerevisiae*, our results and previous reports suggest an opposite, repressive role in the expression of the *FLO* genes and in adhesion-dependent phenotypes. The *C. albicans* Med31, Med20, and Srb9/Med13 contribute to processes highly important for disease: the switch to filamentous morphology and biofilm formation. Moreover, Med31 impacts on virulence in an invertebrate infection model. Our study has implications for understanding the regulation over virulence-associated genes in *C. albicans* and the roles of a key transcriptional regulator in this process.

## Introduction

The transcription factor complex Mediator is associated with RNA polymerase II and it has essential roles in transcription ([1], reviewed in [2]). The yeast Mediator is composed of 25 subunits, which are structurally and functionally organized into four modules [3]–[8]. The core complex is comprised of the Head, Middle and Tail domains [3]–[6]. A fourth, Kinase domain is associated with Mediator under some conditions ([9]–[11]; reviewed in [2]). The core Mediator has a positive role in transcription, while the Kinase domain mainly functions in repression [2].

The roles of Mediator in transcription are complex [2], [12]. Mediator interacts with gene-specific transcription factors and RNA polymerase II and mediates polymerase-activator interactions and formation of the pre-initiation complex (reviewed in [2], [12], [13]). In addition to activated transcription, Mediator also stimulates basal transcription [1], [14], [15]. Further proposed roles for Mediator are in post-initiation steps [12], [16]–[19], re-initiation during multiple rounds of transcription [20] and regulation of chromatin structure [12], [21], [22]. Two recent reports showed an additional role for the core Mediator in sub-telomeric gene silencing [23], [24]. In addition to these versatile roles in gene transcription, Mediator also appears to be a central “integrative hub” for the regulation of gene expression by physiological signals [12]. Examples from yeast include regulation of the Kinase domain by the Ras/PKA pathway via phosphorylation of the Srb9/Med13 subunit [25], and control over the expression of iron-responsive genes by an interplay between the Tail subunit Med2, which has a positive role, and the Kinase domain that phosphorylates Med2 to inhibit its function [7], [26].

The multisubunit Mediator complex emerged early in the evolution of eukaryotes, and the versatility of its functions and its role as an integrative platform for cell physiology could have contributed to the shaping of gene expression programs in different species, for adaptation to specific environments and life styles [27]. Fungi represent an excellent model system for exploring these questions. A comparative analysis in model yeasts *Saccharomyces cerevisiae* and *Schizosaccharomyces pombe* showed remarkable conservation of the roles of Mediator in spite of the fact that these two yeasts are highly divergent [28]. The conserved functions include a broad role in stress responses, and specific, distinct roles of the Mediator domain in the regulation of cell wall dynamics and cell morphology. The Head and Middle domains of Mediator are required for the expression of the cytokinesis genes under the control of the transcription factor Ace2 [23], [28], while the Kinase domain represses transcription of the cell wall adhesins [7], [9], [25], [28], [29]. Furthermore, studies of the Srb11/Ssn8 cyclin subunit of the Mediator Kinase domain in human and plant fungal pathogens (*Cryptococcus neoformans*, *Candida albicans*, *Fusarium vertisilloides* and *Fusarium gramineaurum*) suggest conserved roles in the repression of nutrient responsive functions and genes required for the production of toxins and pigments, as well as a conserved role in stress responses and regulation of cell wall integrity [9], [30]–[37]. Moreover, in *Candida glabrata*, the Mediator Tail subunit Med15/Gal11 plays a conserved role with *S. cerevisiae* in drug resistance mediated by the transcription factor Pdr1 [38], [39].

Here we report the first study of Mediator functions in the human pathogen *C. albicans*. We show that the *C. albicans* Mediator has some conserved functions with *S. cerevisiae* and *Schizo. pombe*, but also has additional roles in the expression of virulence-related genes, most notably the *ALS* adhesins. Phenotypic analysis showed roles for Mediator subunits in phenotypes of *C. albicans* associated with pathogenesis – filamentous growth and biofilm formation. Our data presented here and previous reports [25], [40] show that control of the cell wall adhesins by Mediator subunits Med31 and Srb9/Med13 differs between *S. cerevisiae* and *C. albicans*, suggesting distinct Mediator-dependent control of adhesion in these two yeast species.

## Results

### Roles for the *C. albicans* Mediator Middle domain subunit Med31 in the expression of genes related to morphogenesis, host–pathogen interactions, and pathogen-specific gene families

To start delineating the function of the Mediator complex in *C. albicans*, we made homozygous deletion mutants in the Middle domain subunit Med31. In *C. albicans*, Med31 is encoded by orf19.1429 and it displays 48.2% and 39.80% sequence identity with its *S. cerevisiae* and *Schizo. pombe* orthologs respectively. Transcriptome-wide profiles of the *med31ΔΔ* mutant were obtained and compared to those of a complemented *med31ΔΔ+MED31* strain. Routine manipulations during mutant strain construction can result in gross chromosomal rearrangements, such as aneuploidies [41], which could profoundly affect the results of transcriptome analysis. Inspection of the *med31ΔΔ* transcriptional profile in the chromosomal context did not reveal a colour distribution associated with aneuploidies (Figure S1A), indicating that the *med31ΔΔ* mutant has the same chromosomal structure as the complemented strain. Additionally, gene sets representing 50 kb fragments were included in the Gene Set Enrichment Analysis (see below), and this analysis also did not reveal any gross chromosomal alterations (data not shown).

**Figure 1:**
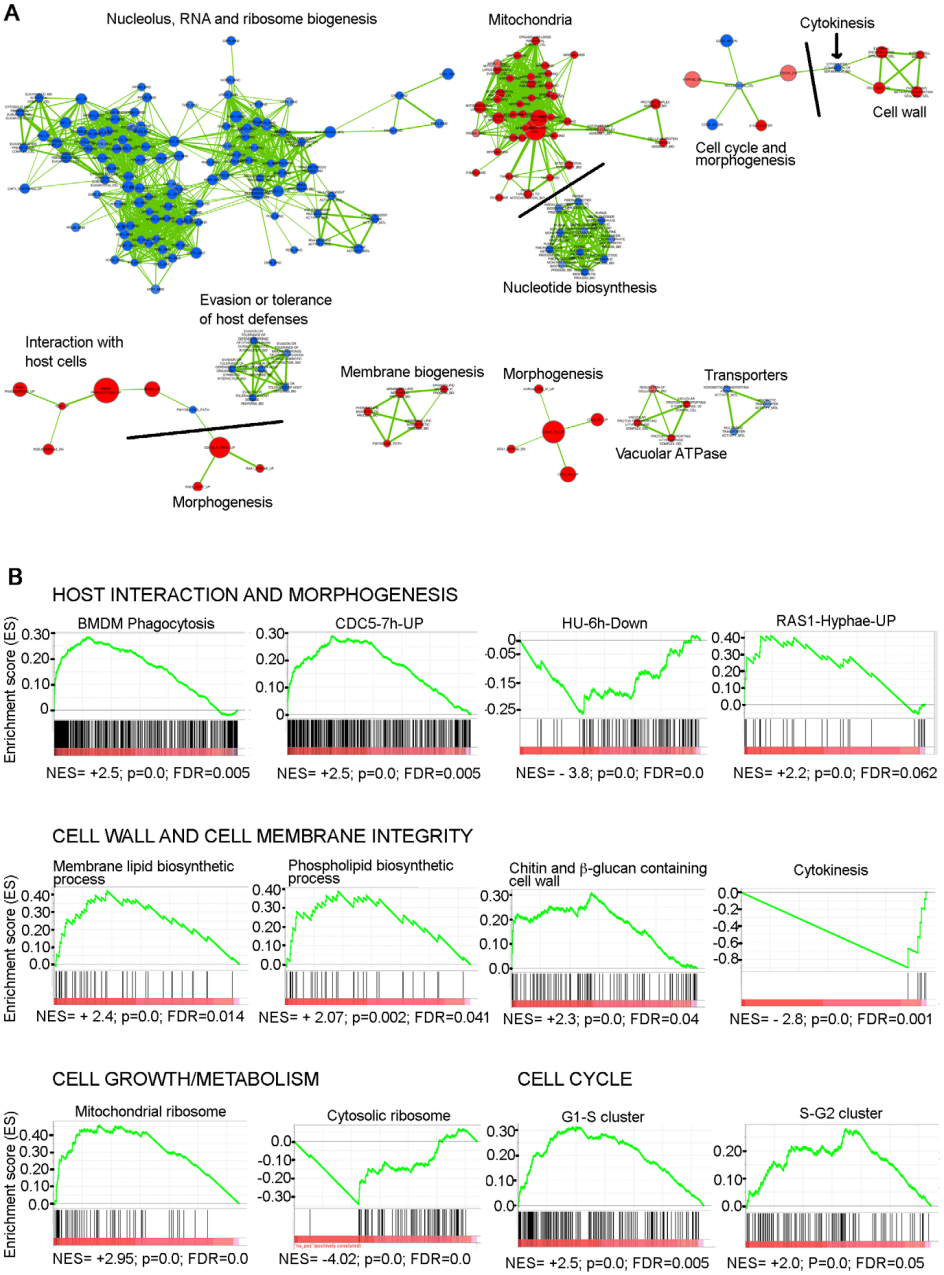
GSEA analysis of the genes differentially expressed in the absence of Med31 in *C. albicans*. A) The network of functional groups of gene regulated by Med31 was constructed with GSEA and Enrichment Map. Blue circles are down-regulated gene sets, while the up-regulated gene sets are represented by red circles. The diameter of the circle reflects the number of modulated gene transcripts in each gene set. Where different functional groups of genes are linked in the network, they are separated by grey lines to indicate the functions. The full network is presented as a pdf document in Figure S2, where the details can be visualized using the “zoom in” function. B) Example enrichment plots for selected genes sets differentially expressed in the *med31ΔΔ* mutant are presented. On the x-axis are genes ranked according to their expression in the *med31ΔΔ* mutant, starting with the up-regulated genes on the left hand side, and all the way down to the down-regulated genes on far right. The position of the individual genes in the gene set are shown by black vertical lines. The cumulative value of the enrichment score (y-axis) is represented by the green line. A positive normalised enrichment score (NES) indicates enrichment in the up-regulated group of genes in the *med31ΔΔ* mutant, while a negative NES indicates prevalence of the genes in the down-regulated group. The title for each of the graphs indicates the genes set used to compare to the *med31ΔΔ* set. BMDM phagocytosis: the set of genes up-regulated in *C. albicans* upon phagocytosis by bone-marrow derived monocytes [47]; CDC5-7h-UP: gene set up-regulated in the *C. albicans* 7 h post depletion of the polo-like kinase *CDC5* [53]; HU-6h-Down: gene set down-regulated upon a 6 h treatment of cells with the DNA replication inhibitor hydroxyurea (HU) [53]; RAS1-Hyphae-up: gene set up-regulated in *ras1* mutants under hyphal growth [52]; G1-S and S-G2: genes expressed at the G1-S and S-G2 phases of the cell cycle in *C. albicans* [54]. p value of 0.0 represents <0.001, FDR is the false discovery rate.

In agreement with a general role for Med31 in gene expression, and consistent with data from *S. cerevisiae* and *Schizo. pombe* [7], [28], [42], [43], 7.8% of the genome (510 genes) was differentially expressed in the absence of *MED31* (cut-off of 1.5 fold, p<0.05, Dataset S1). Out of the genes differentially expressed in *med31ΔΔ* cells, 61.7% (315) were down-regulated and 38.2% (195) were up-regulated. This is consistent with a predominantly positive role of Med31 in transcription, and is in line with reports in *S. cerevisiae* and *Schizo. pombe* [7], [28], [42], [43]. To reveal the cellular pathways regulated by Med31 in *C. albicans*, we performed Gene Set Enrichment Analysis (GSEA) [44], [45]. GSEA compares a list from the transcript profile of interest created by ranking all of the genes according to the change in their expression (in this case that of a *med31ΔΔ* mutant) to a predefined gene set, and asks if a specific gene set is enriched in the top (up-regulated genes) or the bottom (down-regulated genes) of the ranked list [44], [45]. A ranked list of genes from the transcript profile of *med31ΔΔ* cells was compared to a custom database of 8123 gene sets (http://candida2.bri.nrc.ca/andre/GSEA/index.cfm; Sellam and Nantel, submitted) constructed using GO annotations and protein interaction data from CGD (PMID: 19808938), SGD (http://www.yeastgenome.org) and BioGRID [46], most currently published *C. albicans* transcriptional profiling and ChIP-CHIP experiments, our own TF motif database (PMID: 18342603), and *S. cerevisiae* genetic-association data (PMID: 20093466). Since profiles can exhibit correlations with hundreds of overlapping gene sets, significantly enriched gene sets (p<0.005, FDR<25%) were further organized and visualized using the Cytoscape: Enrichment Map plug-in (PMCID: PMC2981572), which produces networks of gene sets that share significant overlaps with each other (Figure 1A shows the most prominent networks of genes; the complete network is shown in Figure S2 where the details can be visualised by using the “zoom in” function in the pdf document). Figure 1B shows examples of enrichment plots for selected gene sets. The complete GSEA output can be found at http://dl.dropbox.com/u/7211133/Med31%20GSEA%20Results.zip.

GSEA detected enrichment for nucleolar functions, rRNA and ribosome biogenesis genes, and genes involved in nucleotide biosynthesis in the set of genes down-regulated in the *med31ΔΔ* mutant, while genes required for mitochondrial function were up-regulated (Figure 1A and 1B). Enrichment was also found in gene sets important for virulence-promoting function in *C. albicans*. Those include genes differentially expressed during *C. albicans*-host interactions with mouse macrophages [47], reconstituted human oral epithelial cells [48] and polymorphonuclear leukocytes [49], as well as genes differentially expressed in conditions which alter cellular morphogenesis, such as the induction of hyphal growth [50], [51], mutations in the Ras-cAMP morphogenesis pathway (*ras1* and *cdc35/cyc1*) [52], and inhibition of cell cycle progression that causes pronounced polarised growth (treatment with hydroxyurea or down-regulation of the polo-like kinase *CDC5*) [53] (Figure 1A and 1B). Genes expressed at the G1-S and S-G2 transition of the cell cycle [54] were up-regulated in the *med31ΔΔ* mutant, as were those required for membrane and cell wall biosynthesis (Figure 1A and 1B). Genes required for cytokinesis were down-regulated (as shown by the black arrow in Figure 1A, and in the enrichment plot in Figure 1B).

Modulation of several gene sets enriched in the *med31ΔΔ* mutant, for example down-regulation of genes required for protein synthesis and up-regulation of those required for cell wall biogenesis, is part of a more general stress response in *C. albicans* [55]. Mediator has been previously implicated in stress responses in yeasts [7], [28], and it is therefore possible that some of the differences in the *med31ΔΔ* transcriptome are due to activation of stress responses upon loss of Med31 function. However, our analysis indicates that this is unlikely to be the cause for much of the differential gene expression in the mutant. There was little correlation between the *med31ΔΔ* transcriptional profile and our large database of transcriptional profiles produced from stressed cells when analysed by GSEA (of note, profile to profiles comparisons such as those done by GSEA tend to produce the strongest correlations and therefore if a correlation existed it is very likely that it would have been detected by GSEA). We further used scatter plots to directly compare the *med31ΔΔ* profile with stressful conditions, such as osmotic or oxidative stress, and these comparisons confirmed lack of extensive correlation between the *med31ΔΔ* transcriptome and differential gene expression upon stress (Figure S1B).

Analysis of gene ontology terms using the GO term finder tool at the *Candida* Genome database and the genes up- or down-regulated by at least 1.5 fold in *med31ΔΔ* cells (see Dataset S1) confirmed that genes related to morphogenesis, mitochondrial function and the cell wall were differentially expressed (Table 1 and Dataset S2). Interestingly, several central regulators of filamentous differentiation, such as the transcription factors Tec1, Efg1, Cph1 and Nrg1, were amongst the down-regulated genes, as were six out of the eight genes from the FGR6 (Filamentous Growth Regulator) family located in the RB2 repeat sequence (Dataset S2). The FGR6 family is one of the gene families found to be enriched in pathogenic yeast species, and specifically expanded in *C. albicans* [56]. While GSEA scored the cell wall gene set as up-regulated, we noticed that there were also several genes in this group that appeared at the bottom of the list, in the down-regulated group. In fact, another *Candida*-specific gene family expanded in pathogens was down-regulated in the *med31ΔΔ* mutant, that encoding the *ALS* cell wall adhesins [56] (Table 2). The major *C. albicans* adhesin *ALS1* was one of the most down-regulated genes in the mutant (5 fold down-regulation, Table 2 and Dataset S1). *ALS5* and *ALS6* were also down-regulated (Table 2), but of note, these genes are expected to be expressed at very low levels in the wild type. Additionally, several other genes encoding cell wall proteins were down-regulated in the mutant, as were genes necessary for cell wall construction and remodelling, in particular those required for cytokinesis and regulated by the transcription factor Ace2 (*e.g.* the chitinase *CHT3* and the endoglucanase *ENG1*) [57] (Table 2; notably GSEA also scored the cytokinesis genes as down-regulated and this is shown in Figure 1). The existence of several down-regulated cell wall genes indicates that the up-regulation of genes with roles in cell wall integrity that is detected in the *med31ΔΔ* mutant (Figure 1 and Table 2) likely reflects a compensatory feedback regulation due to a defective cell wall structure in the absence of Med31. That *med31ΔΔ* mutants have altered cell walls is supported by phenotypic analysis demonstrating changes in sensitivity to the cell wall targeting drugs congo red and calcofluor white (Table 3).

**Table 1:** GO term analysis of genes differentially expressed in the *C. albicans med31ΔΔ* mutant.

GOID	GO term	Cluster frequency (%)	Background frequency (%)	p value	FDR (%)
**Down-regulated genes**					
30447	Filamentous growth	13.6	6.6	0.0031	0
3700	Sequence-specific DNA binding transcription factor activity	5.9	2.1	0.006	0
**Up-regulated genes**					
5761	Mitochondrial ribosome	6.5	1.1	0.00011	0
5739	Mitochondrion	23.7	13.2	0.0082	1.33
30446	Hyphal cell wall	4.3	0.8	0.013	1

Only minimally overlapping GO terms are shown in the Table (p<0.05). The full GO analysis and the list of gene annotated to each of the terms are shown in Dataset S2. FDR is the false discovery rate.

**Table 2:** Cell wall–related genes differentially expressed in the *C. albicans med31ΔΔ* mutant.

Down-regulated genes	
	Gene function
*Known or putative cell wall proteins*	
ALS1	Cell wall adhesin, ALS family
ALS5	Cell wall adhesin, ALS family
ALS6	Cell wall adhesin, ALS family
EAP1	Cell wall adhesin
SCW11	Cell wall protein. Regulated by Ace2.
PIR1	Cell wall protein. Regulated by Ace2.
RHD3	GPI-anchored cell wall protein
PGA26	Putative GPI-anchored protein, adhesin-like
PGA13	Putative GPI-anchored protein, adhesin-like
PGA38	Putative GPI-anchored protein, adhesin-like. Regulated by Ace2.
HYR3	Putative GPI-anchored protein, adhesin-like
HYR10/IFF6	Putative GPI-anchored protein, adhesin-like
*Genes required for cell wall organization and remodeling*	
CHT3	Chitinase. Regulated by Ace2.
CHT1	Chitinase
ENG1	Endoglucanase. Regulated by Ace2.
GSL1	Subunit of ß-1,3 glucan synthase
MNT1	alpha-1, 2 mannosyltransferase, biosynthesis of cell wall mannoproteins
MNN22	Putative alpha-1, 2 mannosyltransferase
RHD1	Putative ß -mannosyltransferase
ECM21	Possible cell wall role
HYM1	Protein of the RAM cell wall integrity signaling pathway, required for cytokinesis
CAS5	Transcription factor required for cell wall integrity

Up-regulated genes	
*Known or putative cell wall proteins*	
PGA31/LDG5	Putative GPI-anchored cell wall protein, Pga30-like, induced during cell wall regeneration
LDG4 (orf19.6484)	GPI family Pga30-like putative cell wall proteins
LDG3	GPI family Pga30-like putative cell wall proteins
PGA1	Putative GPI-anchored protein induced during cell wall regeneration, required for adhesion
EXG2	Cell wall protein, GPI-anchored, similar to *S. cerevisiae* exo-1,3- ß -glucosidase Exg2
ECM331	GPI-anchored protein of the plasma membrane and the cell wall
Orf19.5070	Protein similar to cell wall mannoproteins
Orf19.251	ThiJ/PfpI protein, hyphal cell wall
PGA23	Putative GPI-anchored protein of the cell surface
*Genes required for cell wall organization and remodeling*	
CHS7	Required for chitin synthase activity
CWH41	Processing alpha-glucosidase I, N-linked protein glycosylation, assembly of ß -1,6 glucan
Orf19.7214	Similar to glucan ß -1,3-glucosidase
MNN4-4	Mannosyltransferase
HOC1	Similar to mannosyltransferases
CIS2/ECM38	Putative role in cell wall biogenesis

**Table 3:** Stress responsive phenotypes of the *C. albicans* Mediator mutants.

Conditions affecting membrane integrity				
	*med31ΔΔ*	*ace2ΔΔ*	*med20ΔΔ*	*srb9ΔΔ*
Formamide	++	**−**	+/−	**−**
DMSO	++	**−**	+/−	**−**
nystatin	++	+/−	ND	ND
16°C	+	+	**−**	**−**
**Conditions affecting cell wall integrity**				
Congo red	+	**−**	+/−	++
Calcofluor white	R	R	**−**	+
SDS	++	**−**	+	+
**Other stresses**				
Oxidative stress (H_2_O_2_)	+	**−**	**−**	++
Salt stress (KCl)	++	**−**	**−**	**−**
37°C	**−**	**−**	**−**	**−**
Ethanol	++	**−**	+/−	+

++ very sensitive; + sensitive; +/− mildly sensitive; − wild type phenotype; R resistant; ND not determined.

In conclusion, the transcriptome analysis indicated a broad role for Med31 in cell physiology in *C. albicans*, with functions in morphogenesis and cell cycle progression, growth and metabolism, cell wall integrity, the expression of the cytokinesis genes under the control of the transcription factor Ace2 and those regulated by the interaction of *C. albicans* with host cells. Finally, two gene families enriched in pathogenic yeasts, the *FGR* family of filamentous growth regulators and the *ALS* adhesins, required Med31 for wild type expression levels.

### Regulation of Ace2-dependent genes and cell wall adhesins by Med31 in yeast and hyphal growth

Down-regulation of Ace2 target genes in the absence of Med31 in *C. albicans* is in agreement with a role for Med31 and other Mediator subunits in Ace2-dependent gene expression that is conserved between *C. albicans*, *Schizo. pombe* and *S. cerevisiae* (this study and [28]). Comparing more broadly the genes affected in *med31ΔΔ* cells with those reported to be differentially expressed in the *C. albicans ace2* mutant [57] revealed that differential expression of 35 genes is shared between these two transcription factors (Table S1). Genes involved in cytokinesis and cell wall functions were predominant in the shared “down-regulated” group, whereas mitochondrial biogenesis genes were predominant in the shared “up-regulated” group (Table S1). This analysis suggests that the functions of Med31 in cell wall integrity and metabolism are mediated, at least in part, by Ace2-dependent roles.

**Figure 2:**
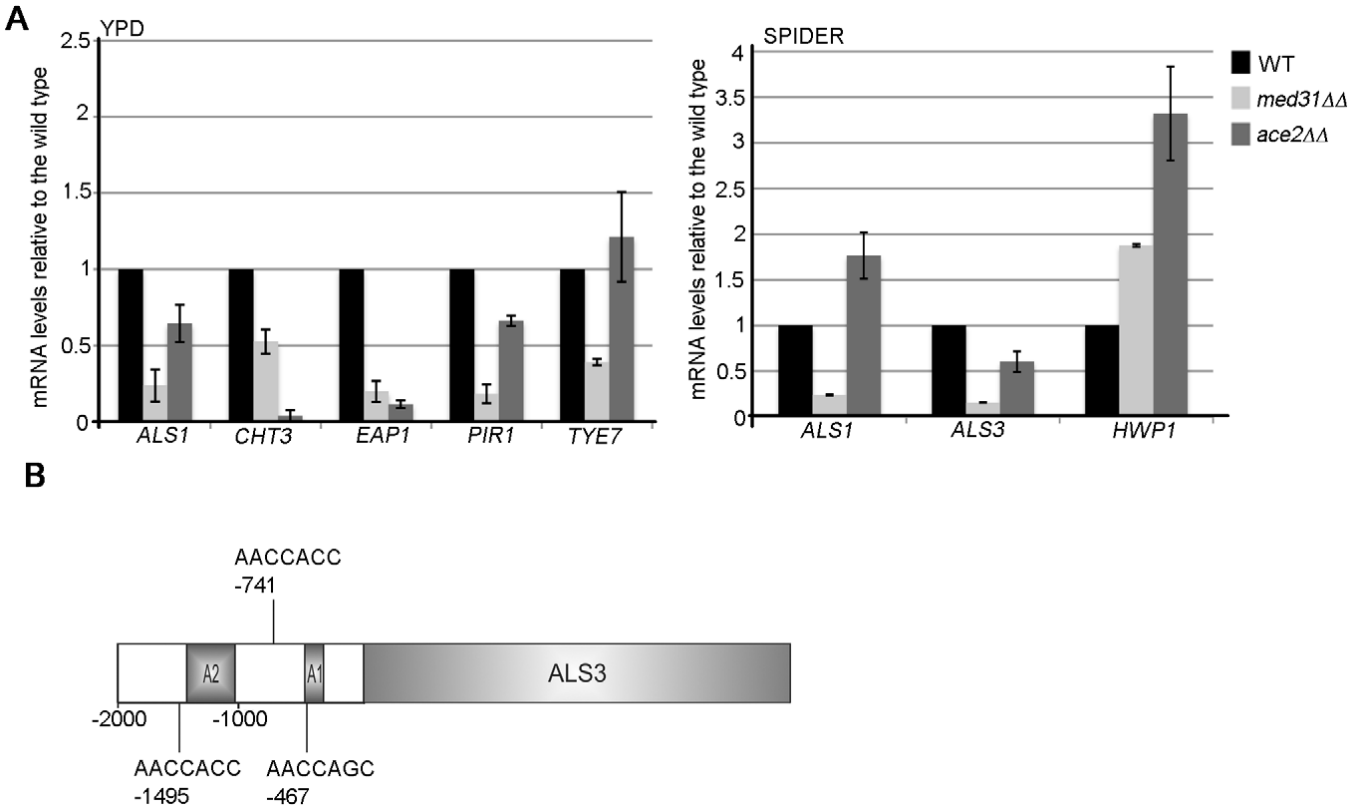
Med31 is required for the expression of Ace2-dependent genes and adhesins in yeast and hyphal growth. A) Cells from *med31ΔΔ* or *ace2ΔΔ* mutants were grown in YPD at 30°C (yeast morphology) or in Spider media at 37°C to induce filamentous growth, and levels of the indicated genes determined by quantitative PCR. The expression of the indicated genes was normalised to *ACT1* and expressed relative to wild type levels, which were set to 1. Equivalent results were obtained when the glyceraldehyde-3-phosphate dehydrogenase (GAPDH) encoding gene *TDH3* was used for normalization (Figure S3). Shown are the averages of at least three independent experiments and the standard error. B) The promoter regions (2 kb) of *ALS1*, *ALS3* and *EAP1* were searched for putative Ace2 binding motifs from *S. cerevisiae* (RRCCAGC) or *C. albicans* (MMCCASC) [57]. The three motifs conforming to *C. albicans* consensus sequences were found for the *ALS3* promoter, whereas none were found for the other two genes. The activating regions A1 and A2 in the *ALS3* promoter were mapped by [59]: A1: from −321 bp to −471 bp (upstream of the START codon); A2: from −1049 to −1438 bp.

To explore this further, we used quantitative PCR (qPCR) to directly compare the expression levels of candidate genes in the *med31ΔΔ* and *ace2ΔΔ* mutants (Figure 2A). Under yeast growth conditions (as was done for the transcriptome analysis) the mRNA levels for the chitinase *CHT3* and the cell wall proteins *PIR1*, *EAP1* and *ALS1* were reduced in both *med31ΔΔ* and *ace2ΔΔ* cells (albeit to a different degree). *CHT3* and *PIR1* have been previously reported as Ace2-targets, while *ALS1* and *EAP1* were not [57]. The expression of the transcription factor *TYE7* was reduced in *med31ΔΔ* cells (consistent with the microarray data), but not in the *ace2ΔΔ* mutant.

In *C. albicans*, the expression of cell wall proteins is activated upon filamentous differentiation [50], [51], [58]–[61]. This includes *ALS1* and other *ALS* and non-*ALS* adhesins. Therefore, we next tested if Med31 was required for the expression of the *ALS1*, and two hypha-specific adhesins *ALS3* and *HWP1*, during filamentous growth. For these experiments cells were grown in filament-inducing Spider media at 37°C. The mRNA levels of both *ALS1* and *ALS3* were down-regulated in *med31ΔΔ* cells (Figure 2A). Ace2 was not required for *ALS1* expression during hyphal growth, while the levels of *ALS3* were lower in cells lacking Ace2, but the effect was less than in the absence of Med31 (Figure 2A). *HWP1* was up-regulated in both mutants (1.8–3 fold) (Figure 2A). Collectively, the qPCR analysis suggests that Med31 and Ace2 have common, but also independent roles in the expression of the cytokinesis genes and the cell wall adhesins during yeast and hyphal growth.

Given that we found novel cell wall protein targets that require Ace2 for wild type expression (*ALS1*, *ALS3* and *EAP1*), we searched the upstream regulatory regions of these genes for putative Ace2 binding sites as defined in [57]. There are three Ace2-binding motifs within 1.5 kb upstream of the start codon for *ALS3* (Figure 2B). No motifs that strictly conform to the consensus sequence were found in the promoters of *ALS1* and *EAP1*, although variant motifs could be found (data not shown). The motifs in the *ALS3* promoter included one at −467 bp, which is in a region found to be essential for *ALS3* activation in hyphae (the so-called A1 region) [59]. This suggests that *ALS3* could be a direct target of Ace2.

### Phenotypic analysis shows roles for Med31 in cytokinesis, morphogenesis, and virulence

**Figure 3:**
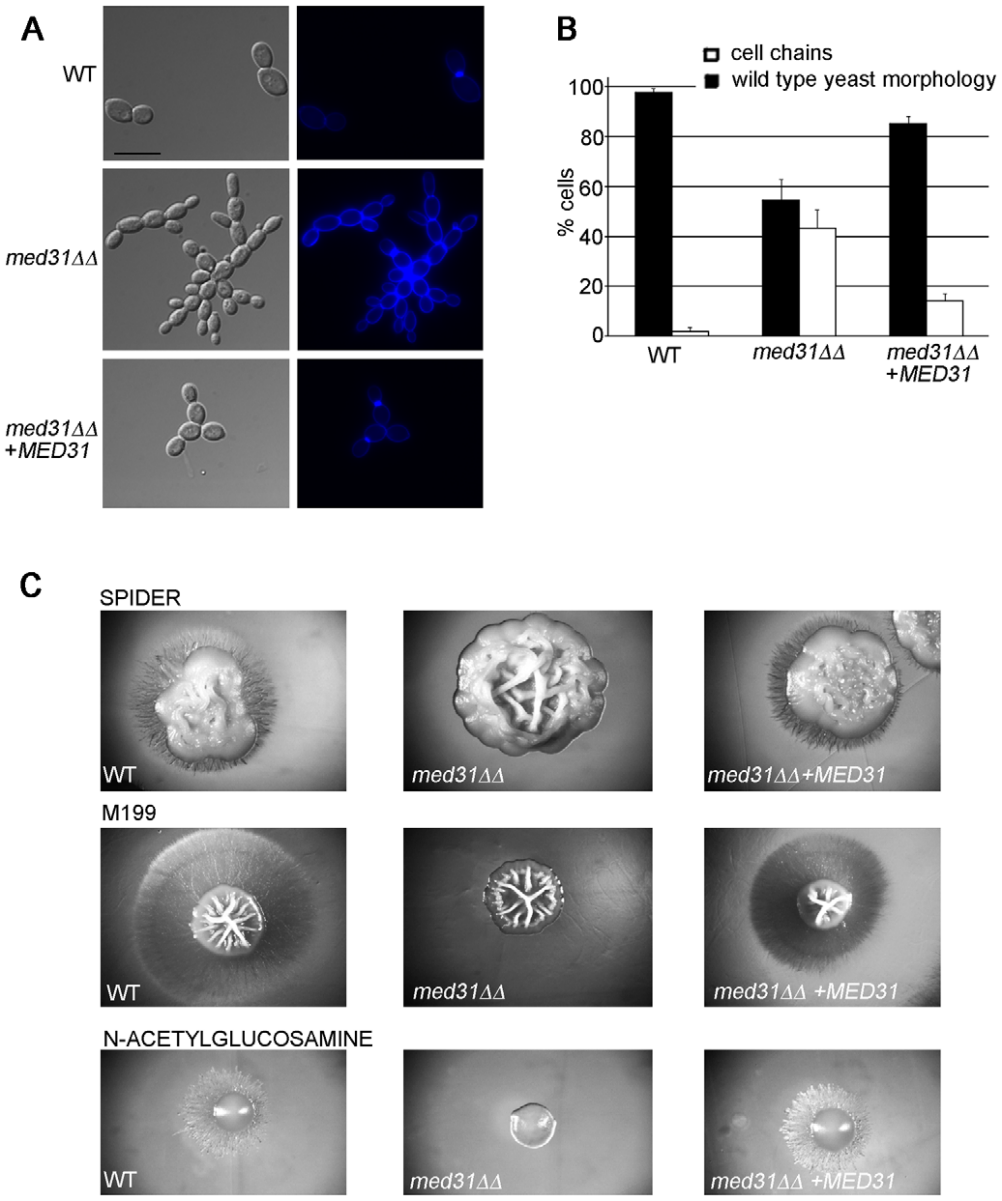
Med31 is required for cytokinesis and filamentous growth of *C. albicans*. A) Cultures of wild type C. *albicans*, the *med31ΔΔ* mutant and the complemented *med31ΔΔ*+*MED31* strain were grown to log phase and cells were observed by microscopy using DIC for bright field (left panel) or through the DAPI filter for calcofluor white staining (right panel). Strains lacking Med31 display a cell separation defect, forming cell chains with the cells attached at the mother-daughter junction, as judged by staining with calcofluor white. B) To determine the proportion of cell chains at least 200 cells were counted for each of the strains. Cell counts were performed after a brief 1 s sonication to disperse cell aggregates. Shown are averages of three independent experiments and the standard error. C) Wild type or mutant strains were streaked on plates containing filamentation-inducing media and incubated at 37°C for 4–5 days. The colonies were photographed using a stereo dissecting microscope. The *med31ΔΔ* mutant was unable to undergo filamentous differentiation on plates in all media tested. The mutant was also compromised for filamentation in liquid media (data shown in Figure S5).

We next performed phenotypic analysis of the *C. albicans med31ΔΔ* mutant to address the biological relevance of the observed changes in gene expression. Cells lacking Med31 displayed a cytokinesis defect (Figure 3A), consistent with a role in Ace2-dependent gene expression. ∼40% of cells from *med31ΔΔ* cultures showed a cell-chain phenotype typical of mutants that cannot undergo cytokinesis. This phenotype was observed in two independently constructed homozygous deletion mutants and was partially complemented by re-introduction of a wild type copy of *MED31* into the mutant genome (Figure 3A and 3B).

The *med31ΔΔ* mutant also displayed phenotypes consistent with altered cell membrane and cell wall integrity that were suggested by transcriptome analysis. The mutant was sensitive to formamide, SDS, the sterol-binding antifungal drug nystatin, DMSO and growth at 16°C, all phenotypes consistent with defective membrane structure. The mutant was also sensitive to the cell wall-targeting drug congo red, but more resistant to the chitin-binding dye calcofluor white (Table 3 and Figure S4). Furthermore, the *med31ΔΔ* mutant was sensitive to oxidative and salt stress and ethanol (Table 3 and Figure S4). Some of these phenotypes are also observed in *S. cerevisiae* and *Schizo. pombe med31* mutants, suggesting conserved roles [28], [43]. We also tested the *C. albicans ace2ΔΔ* mutant side by side with *med31ΔΔ* for tolerance to the various compounds (Table 3 and Figure S4). Resistance to calcoflour white was also observed in the *ace2ΔΔ* mutant (Table 3 and [62]), as was a mild sensitivity to nystatin and growth at 16°C, indicating that these phenotypes could be due to the role of Med31 in Ace2-dependent gene expression. However, the other sensitivities of the *med31ΔΔ* mutant were not shared by the *ace2ΔΔ* mutant, and are therefore unrelated to Ace2-dependent phenotypes.

**Figure 4:**
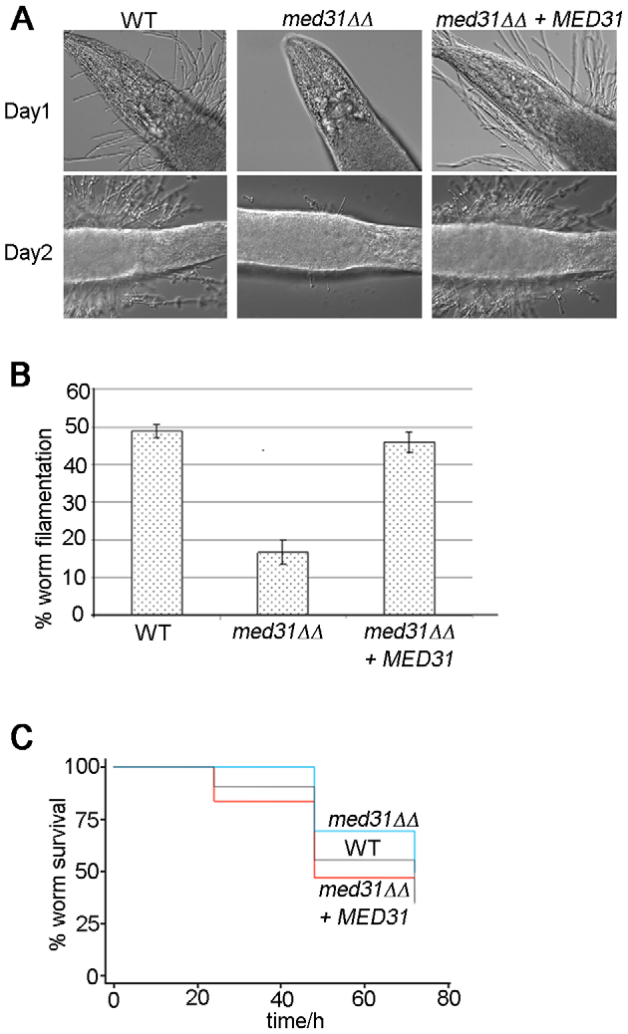
The *med31ΔΔ* mutant is defective for filamentation and virulence in an animal host. A) The worm *C. elegans* was infected by wild type *C. albicans*, the *med31ΔΔ* mutant and the *med31ΔΔ*+*MED31* complemented strain and the appearance of penetrative filamentation was monitored daily over a period of seven days. The worms were photographed with a 40× magnification objective. B) The percentage of worm filamentation was determined after three days of infection. Shown are averages of 4 experiments and the standard deviation. The p value was <0.002 for both the comparison of the mutant with the wild type, and the mutant with the complemented strain. C) The ability of the *med31ΔΔ* mutant to kill worms was determined in the first 80 h post infection, when most of the worm death due to penetrative filamentation occurs. Three independent experiments were performed and equivalent results obtained. A representative experiment is shown. The *med31ΔΔ* mutant killed the *C. elegans* host with delayed kinetics compared to the wild type and the reconstituted strains (blue line- *med31ΔΔ*, grey line- WT, red line- *med31ΔΔ*+*MED31*). The log-rank test used for statistical analysis returned a p value of 0.0032 for the wild type versus *med31ΔΔ* mutant comparison, while there was no significant difference between the wild type and the *med31ΔΔ*+*MED31* complemented strain p = 0.5572).

The transcriptome analysis indicated a role for Med31 in cellular morphogenesis and we therefore tested whether Med31 was necessary for the yeast-to-hypha morphogenetic switch in response to a variety of inducers *in vitro*. The *med31ΔΔ* mutant was unable to produce filaments on solid Spider and M199 media, or on plates containing N-acetylglucosamine (Figure 3C). The mutant was also compromised for filamentation in liquid media, however to a lesser extent than on plates (Figure S5). *med31ΔΔ* cells could filament in response to serum and in M199 media in culture, but with delayed kinetics and with a proportion of cells remaining in yeast form (Figure S5). In Spider media the mutant had a more pronounced defect, and even after 7 h cells were still largely in yeast form (Figure S5). However, after prolonged incubation of 12 h, filaments were observed in this medium also (data not shown). To address whether the role for Med31 in filamentous growth would be important in a disease context, we further tested the ability of *med31ΔΔ* cells to filament *in vivo*, using the *C. albicans*-*Caenorhabditis elegans* infection model [63], [64]. This is a well-established host-pathogen system, which recapitulates key elements of disease as seen in vertebrates, most notably, the requirement for filamentous growth [63]. Only 16.5% of the worms infected with the mutant developed filaments after 3 days compared to 49% for worms infected with wild type *C. albicans* or the complemented strain (Figure 4A and 4B). Moreover, for those *med31ΔΔ*-infected worms that did develop filaments, there was a significant delay in filamentation, there were many fewer filaments per worm compared to the wild type or complemented strains, and the filaments were much shorter (Figure 4A; a similar phenotype was observed even after 7 days of infection, Figure S6). We also evaluated the pathogenic potential of the *med31ΔΔ* mutant in the worm. The kinetics of killing of the worm by *C. albicans* was delayed in worms infected with the *med31ΔΔ* mutant compared to those infected with the wild type (p<0.02 for all experiments performed) (Figure 4C). This result supports a role for Med31 in *C. albicans* virulence.

### Med31 is necessary for biofilm formation, and *ALS1* is a relevant gene target for this phenotype

**Figure 5:**
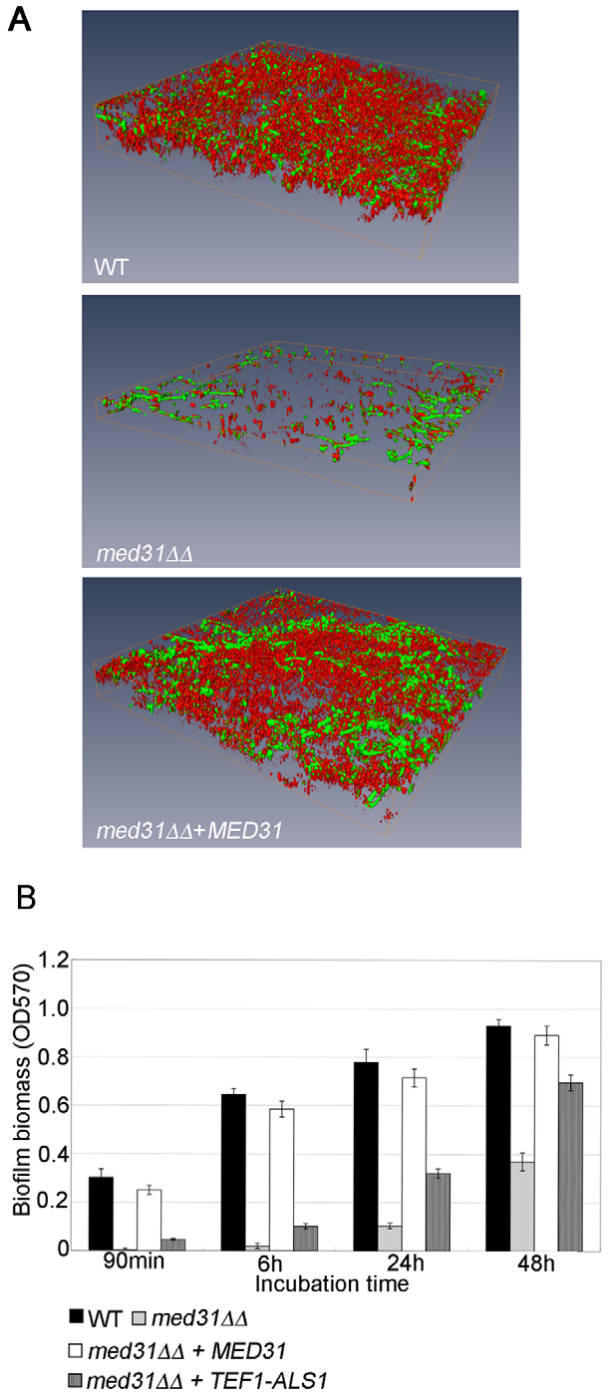
Med31 is necessary for wild-type biofilm formation, and *ALS1* is a relevant Med31 gene target for this phenotype. A) Mature biofilms (48 h) formed on serum-coated silicone disks were stained with FUN-1 and concavalin A-Alexa488 and imaged using confocal microscopy. The 3D images were reconstructed using the Amira 5.2.1 software. Cells lacking *MED31* showed a clear defect in their ability to form mature biofilms. The depths for the biofilms were as follows: WT = 54.57 µm, *med31ΔΔ* = 23.47 µm and *med31ΔΔ+MED31* = 45 µm. A biofilm formation defect for the *med31ΔΔ* mutant was confirmed by scanning electron microscopy (Figure S7). B) Biofilms of the indicated strains were formed in 96 well plates and analyzed quantitatively by crystal violet staining over a 48 h time course. Shown are averages of at least three experiments performed in triplicates and the standard error. p values were <0.01.

We next sought to show that the regulation of the adhesins by Med31 was biologically relevant. To that end, we tested the ability of the *med31ΔΔ* mutant to form biofilms, as both *ALS1* and *ALS3* are key adhesins for biofilm formation by *C. albicans* [65]. Biofilm formation was tested *in vitro*, on serum-coated silicone disks as previously described [66]. In the absence of Med31, biofilms were severely compromised in both density and depth, as determined by confocal scanning laser microscopy (Figure 5A). Scanning electron microscopy (SEM) showed a similar defect (Figure S7). Quantitative analysis confirmed the phenotype observed by microscopy (Figure 5B). The *med31ΔΔ* mutant displayed a strong defect at the earliest time point of 90 minutes (adherence stage). The *med31ΔΔ* mutant grew somewhat slower than the wild type in planktonic conditions (Figures S4 and S8), however at the 90 minutes time point in the biofilm formation assay we did not observe significant cell growth for any of the strains, including the wild type (data not shown), strongly suggesting that the defect in the *med31ΔΔ* mutant is due to an adherence defect and not to the observed slower growth.

Rescue by over-expression of target genes has been previously used as a strategy for identification of transcription factor targets relevant for biofilm formation (for example in the *C. albicans bcr1* mutant [65]). We employed a similar strategy to test whether *ALS1* is a relevant Med31 target gene for biofilm formation. The idea is that, if lower expression of *ALS1* in the *med31ΔΔ* mutant is contributing to the biofilm defect, its ectopic expression under a constitutive promoter should, at least in part, rescue biofilm formation by *med31ΔΔ* cells. As shown in Figure 5B, expression of *ALS1* in the *med31ΔΔ* mutant under the constitutive *TEF1* promoter led to a substantial rescue of the biofilm defect at all time points, including the early adherence and initiation stages. Similar rescue was observed in three independent *med31ΔΔ+TEF1-ALS1* clones. Expression of *TEF1*-*ALS1* in the wild type strain did not significantly change biofilm biomass (data not shown). As an independent confirmation that the biofilm formation defect of the *med31ΔΔ* mutant is not due to slower growth, we tested whether introduction of the *TEF1-ALS1* construct was rescuing the growth defect of the *med31ΔΔ* mutant. We did not observe rescue of the mutant growth defect by *TEF1-ALS1* (Figure S8), although biofilm formation was rescued (Figure 5B). This confirms that the biofilm defect is due to lower adherence and not slower growth. Collectively, these results suggest that *ALS1* is a biologically relevant gene target of Med31 for biofilm formation by *C. albicans*.

### The Mediator Head domain subunit Med20 and Srb9/Med13 from the Kinase domain also contribute to morphogenesis, biofilm formation, and adhesin gene expression in *C. albicans*

**Figure 6:**
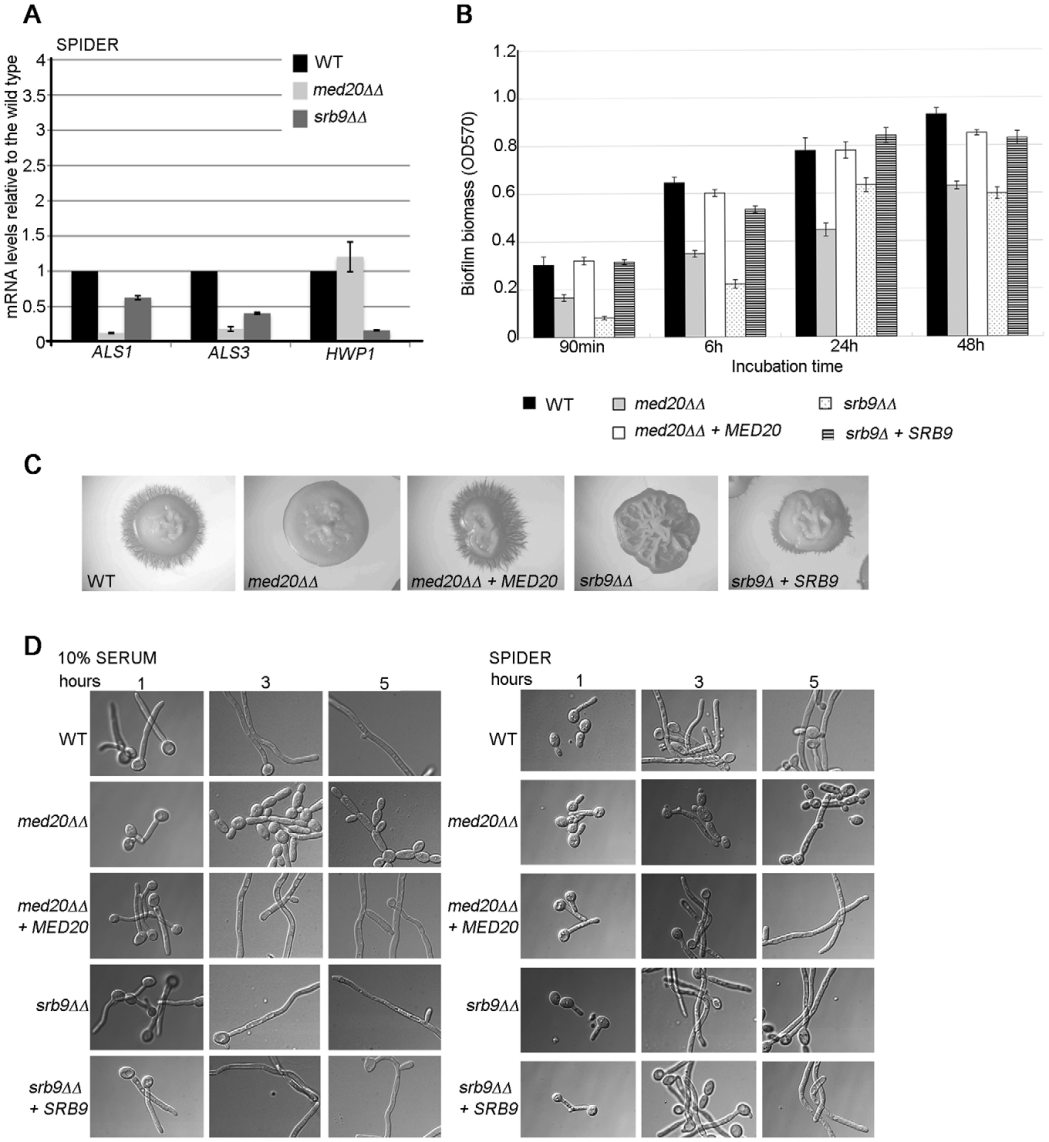
The Mediator Head domain subunit *MED20* and *SRB9/MED13* from the Kinase domain contribute to filamentous growth and biofilm formation by *C. albicans*. A) Cells from the indicated strains were grown in hyphae (Spider media) and relative mRNA levels for the indicated genes determined by qPCR. *ACT1* was used for normalization. Similar results were obtained when *THD3* was used as the normalization control (Figure S3). Shown are averages for three independent biological repeats and the standard error. B) Biofilm formation for the wild type, *med20ΔΔ*, *srb9/med13ΔΔ* and complemented strains was assessed in 96 well plates by crystal violet staining as in Figure 5B. Shown are averages of at least three independent biological replicates and the standard error. p values were <0.05. C) Filamentous growth on Spider plates was assessed after 4–5 days at 37°C. The *med20ΔΔ* and *srb9/med13ΔΔ* mutants were defective, and the phenotype was complemented by reintroducing *MED20* or *SRB9/MED13* into the respective mutant genomes. D) Overnight cultures were grown in YPD to saturation and cells diluted into pre-warmed filamentation media (YPD +10% serum or Spider media) to assess filamentous growth at 37°C. Photographs were taken after 1, 3 or 5 hours.

**Figure 7:**
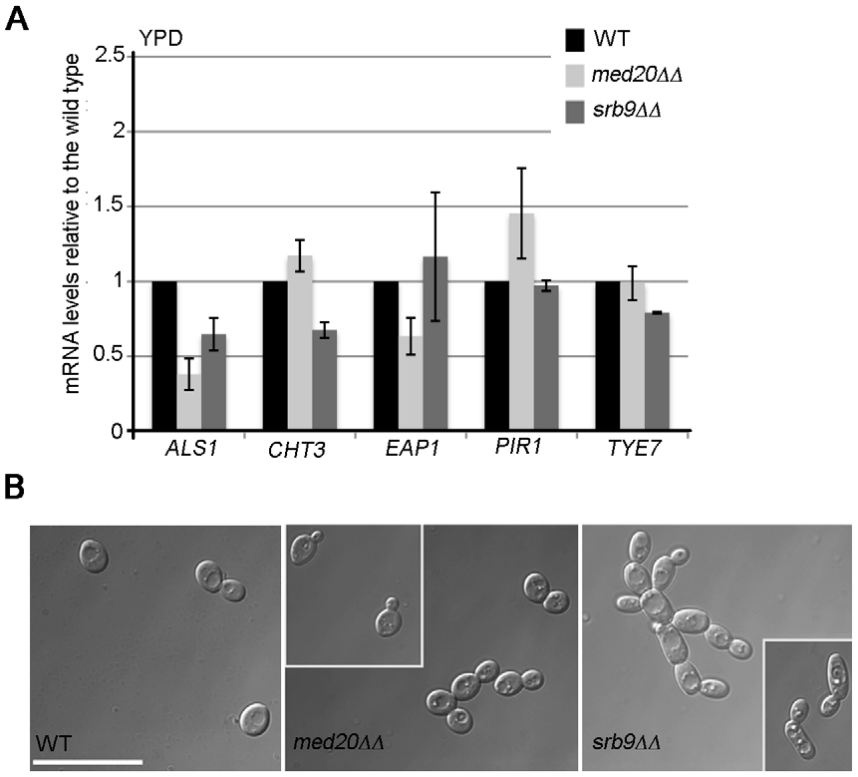
Regulation of adhesins and Ace2-dependent genes by *MED20* and *SRB9/MED13* in *C. albicans*. A) Cells from the indicated strains were grown in yeast form (YPD) and relative mRNA levels for the indicated genes determined by qPCR. *ACT1* was used for normalization. Similar results were obtained when *THD3* was used as the normalization control (Figure S3). Shown are averages for three independent biological repeats and the standard error. B) Yeast cell morphology of the wild type and Mediator mutants was assessed in YPD at 30°C. The scale bar is 20 µm.

To address more generally the roles of Mediator in *C. albicans*, we constructed mutants in the Med20 subunit from the Head domain (orf19.2711.1) and the Kinase domain subunit Srb9/Med13 (orf19.1452; for simplicity the mutant is indicated as *srb9ΔΔ* in the figures) [27]. In agreement with a general role for Mediator in adhesin gene expression, both *MED20* and *SRB9/MED13* were required for the expression of *ALS1* and *ALS3* in hyphae, and *SRB9/MED13* was further required for wild type transcript levels of *HWP1* (Figure 6A). Consistent with the effects on adhesins, both *MED20* and *SRB9/MED13* were necessary for wild type biofilm formation (Figure 6B). In regards to filamentous growth, we observed a similar trend as with the *med31ΔΔ* cells: filamentous growth was severely compromised in *med20ΔΔ* and s*rb9/med13ΔΔ* mutants on plates (Figure 6C), while in liquid media the effects were much less pronounced (Figure 6D). In liquid media, *med20ΔΔ* showed a mild defect with a larger proportion of pseudohyphae (for example see the 3 and 5 h time points in serum), while *srb9/med13ΔΔ* behaved like the wild type (Figure 6D). To address the similarities and differences between the Mediator subunits more broadly, we analysed whether Med31-dependent genes during yeast growth were also dependent on Med20 and Srb9/Med13 for their expression. *EAP1* was down-regulated in *med20ΔΔ* cells, *ALS1* was down-regulated in both mutants, and *CHT3* was only affected in *srb9/med13ΔΔ* cells (Figure 7A). The *med20ΔΔ* mutant did not display a cytokinesis defect, consistent with wild type *CHT3* levels (Figure 7B). The *srb9/med13ΔΔ* cells were slightly elongated and a fraction (between 20–40%) formed what appeared similar to pseudohyphae, perhaps consistent with lower *CHT3* levels (Figure 7B). The *med20ΔΔ* and *srb9/med13ΔΔ* mutants shared some (but not all) sensitivities to antifungal compounds and stress conditions with the *med31ΔΔ* strain (Table 3), and some of these sensitivities are conserved with what has been reported for the homologous mutations in model yeasts [28].

### The roles of Med31 in adhesion are different between *S. cerevisiae* and *C. albicans*

**Figure 8:**
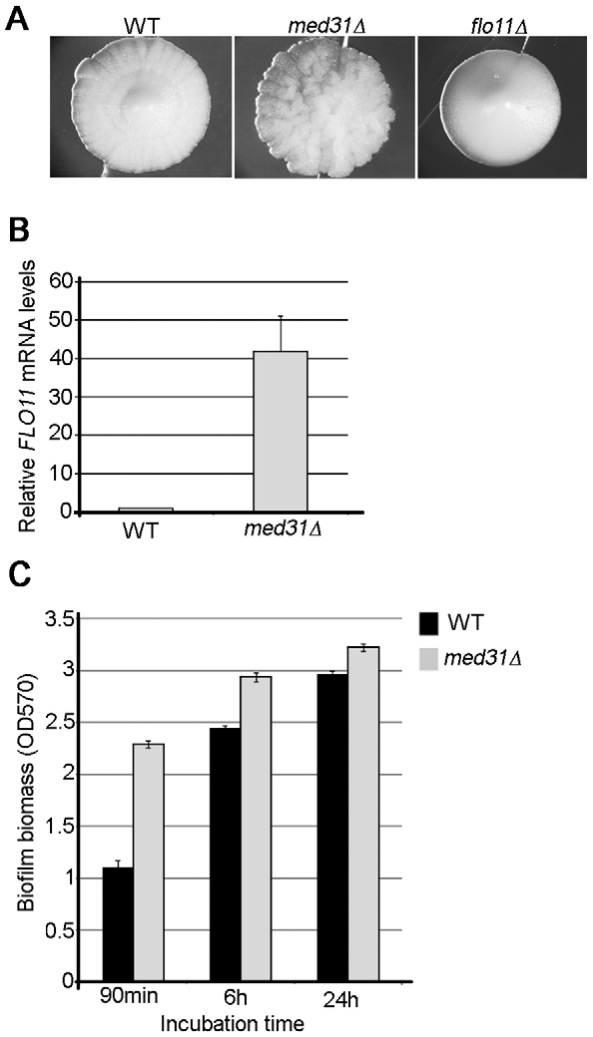
The roles of Med31 in adhesion are different between *S. cerevisiae* and *C. albicans*. A) Colonies of wild type *S. cerevisiae* Σ1278b and the *med31Δ* mutant were grown on YPD plates at 30°C and photographed. The *flo11Δ* strain was used as the negative control, to show smooth colony morphology in the absence of *FLO11*. B) Expression of *FLO11* was tested by qPCR after 90 min in 0.2% glucose synthetic complete media, the condition used for biofilm formation in C). Levels of *FLO11* were normalized to *ACT1*, and expressed related to the wild type, which was set to 1. Shown are averages from at least three independent biological repeats and the standard error. C) The ability of the *S. cerevisiae med31Δ* mutant to adhere to polystyrene was assessed in 0.2% glucose synthetic complete media as described in Materials and Methods. Quantification was performed by crystal violet staining. At least three independent cultures were used, assayed in quadruplicates. For the mutant, two independently constructed deletion strains were used and gave equivalent results. The *flo11Δ* mutant was assayed in parallel as a negative control and showed no adherence at any of the time points (not shown). p<0.001.

In the model yeast *S. cerevisiae* Mediator has been implicated in the repression of the *FLO* cell wall adhesins, due to the inhibitory functions of the Kinase domain subunits (including Srb9/Med13) and the Tail domain component Sin4/Med16 [25], [40]. Repression of the cell wall adhesins by the Mediator Kinase domain has also been reported in *Schizo. pombe* [28]. Our data in *C. albicans* showed that Srb9/Med13 is a positive regulator of the genes encoding adhesins (Figure 6), thus indicating that the roles of this Mediator Kinase domain subunit in adhesion are different in *C. albicans*. To probe this notion further, we tested the role of Med31 in adhesion in *S. cerevisiae* using the Σ1278b strain background, which expresses the *FLO11* adhesin and is capable of adhesion-dependent phenotypes such as biofilm formation on polystyrene [67]. The wrinkled colony morphology in the *med31Δ* mutant was enhanced compared to the wild type, a phenotype that is indicative of higher *FLO11* levels (Figure 8A). Indeed, the expression of *FLO11* was significantly up-regulated in the absence of *MED31* (40 fold), and consistently, the mutant was hyper-adherent in the *S. cerevisiae* biofilm model (Figure 8B and 8C). The levels of the other *FLO* family members, which are silent in the wild type, were not up-regulated under the conditions assayed (data not shown). These results are consistent with a repressive role for Med31 in the expression of *FLO11* and biofilm formation in *S. cerevisiae*.

## Discussion

So far, most comparative analysis of transcription factor function in fungi have focused on DNA binding gene-specific transcription factors, and only very few studies have addressed the functions of transcriptional co-regulator complexes, such as Mediator. With Mediator, conserved roles have been reported for the core complex and the Kinase domain, including distinct roles of the sub-domains in cell wall dynamics shared by the divergent species *S. cerevisiae* and *Schizo. pombe* [9], [23], [25], [28], [29]. Our study is the first comprehensive analysis of Mediator components in the pathogen *C. albicans*. We identified conserved functions for Mediator subunits in Ace2-dependent gene expression and stress responsive phenotypes with the model yeasts, but also novel roles in the expression of genes related to virulence attributes of *C. albicans*. In particular, our study uncovered roles in the expression of the cell wall adhesins and functions in biofilm formation, which are shared between the Middle domain subunit Med31, the Head domain subunit Med20 and Srb9/Med13 from the Kinase domain.

### Overlapping and specific roles for the Mediator subunits in *C. albicans*

Our data in *C. albicans* is consistent with reports in model yeasts in that Mediator subunits show overlapping, but also some specific roles [7], [28], [43]. In *S. cerevisiae* and *Schizo. pombe*, mutants in the Head and Middle domain subunits tend to correlate in regards to gene expression and cellular phenotypes [7], [28], [43]. We also find this for the *C. albicans med31ΔΔ* and *med20ΔΔ* mutants. For example, the expression of the cell wall adhesins *ALS1*, *ALS3*, *EAP1* is down-regulated in the absence of either *MED31* or *MED20*, both mutants display biofilm defects and a stronger filamentation defect on plates than in liquid media, and they share sensitivities to compounds such as formamide, DMSO, SDS, ethanol and congo red (Table 3). However, our data also supports differences in the roles of Med20 and Med31: Med20 is not required for the expression of the Med31-regulated genes *CHT3*, *PIR1* and *TYE7*, it shows a milder biofilm formation and filamentous growth defect than Med31, and there are differences in the sensitivities to some compounds between the two mutants. Shared but also distinct roles of Med20 and Med31 are supported by results in *S. cerevisiae* showing a 0.41 Pearson correlation coefficient between the transcript profiles form the two mutants [43]. In *Schizo. pombe*, in addition to Med31, Med20 and other Head domain subunits also control the expression of Ace2-dependent genes and the mutants show defects in cell separation [28]. Deletion of *MED20* in *C. albicans* did not result in lower expression of *CHT3* or a cytokinesis defect (Figure 7). Together with Med18 and the C-terminal domain of Med8, Med20 forms a structural and functional sub-complex within the Head domain, the Med8C/18/20 submodule [28], [68], [69]. Genetic analysis in *Schizo. pombe* supports the idea that Med18 can compensate for the loss of Med20 [28]. It is therefore possible that Med18 would need to be inactivated in *C. albicans* to uncover the roles of the Mediator Head domain in Ace2-dependent transcription and cytokinesis.

In contrast to the core Mediator complex which functions predominantly in transcriptional activation, the Kinase domain is mainly a repressor of transcription and the phenotypes of the Kinase mutants in model yeasts tend not to correlate with mutants in the core Mediator (for example see [7], [28]). Our data in *C. albicans* supports this, as the *med31ΔΔ* and m*ed20ΔΔ* mutants were more similar to each other in terms of gene expression and phenotypes than to the *srb9/med13ΔΔ* strain. However, the *srb9/med13ΔΔ* strain shared some phenotypes with *med31ΔΔ* and m*ed20ΔΔ* including the effects on *ALS* gene expression, biofilm defects and sensitivities to compounds that affect the cell wall, such as congo red and SDS. The Mediator kinase domain consists of four subunits: Srb8/Med12, Srb9/Med13 and the kinase-cyclin pair Srb10/Srb11. The *C. albicans* mutants in Srb10/Srb11 were in the Kinase collection constructed by Blankenship et al. [35]. Our *srb9/med13ΔΔ* mutant shares the sensitivity to oxidative stress with cells inactivated for *SRB10* or *SRB11* (Table 3 and [35]), however the *srb10* and *srb11* mutants were reported to display wild type biofilm formation [35]. The Kinase domain subunits share many functions, but can also have different roles, in particular positive functions in the transcription of some genes have been reported that are not shared by the whole domain [70], [71]. The data reported here (Figure 6) and in [35] support distinct roles for the *C. albicans* Mediator Kinase domain subunits in biofilm formation.

### The connection between Med31 and Ace2

Our data indicates that in *C. albicans* the transcriptional activator Ace2 could be modulating a number of Med31-dependent effects on gene expression, in particular transcriptional activation of the cytokinesis genes, the expression of the adhesins *EAP1* and *ALS3* and the cell wall protein *PIR1*, and the regulation of genes with mitochondrial functions (Figure 2 and Table S1). The *ALS3* promoter contains a putative consensus Ace2 binding site within a region known to be required for activation [59], suggesting it could be a direct target of Ace2. Consistent with shared functions, both *ace2ΔΔ* and *med31ΔΔ* mutants display a cytokinesis defect, as well as adherence and biofilm formation phenotypes (this study and [72]). In *Schizo. pombe* Mediator interacts directly with Ace2, via the Head domain subunit Med8, and therefore Mediator plays a direct role in Ace2-dependent transcription [73]. By analogy to *Schizo. pombe*, and given that we did not observe a change in transcript levels for *ACE2* in the *med31ΔΔ* mutant that would indicate an indirect effect (Dataset S1), we propose that in *C. albicans* Ace2 also interacts directly with Mediator for transcriptional activation. Our data also suggests that Med31 and Ace2 have roles in transcription that are independent of each other. Unlike Ace2, which is indispensable for cytokinesis in *C. albicans*, a milder phenotype is observed in the absence of Med31 (Figure 3B). There is only a partial overlap between genes differentially expressed in *ace2* and *med31* mutants respectively ([57] and this study, Table S1), and the degree of the effects on gene transcription differs between the two mutants (for example *CHT3* is much more affected by inactivation of *ACE2* than *MED31*, while *MED31* has a stronger effect on *PIR1*, Figure 2). *ALS1*, which we show is a key target of Med31, does not require Ace2 during high transcription in hyphae (Figure 2). As in *Schizo. pombe* [28], [73], it could be that in *C. albicans* Mediator subunits additional to Med31 are involved in co-activating Ace2-dependent transcription. Conversely, Med31 certainly acts through additional DNA binding transcription factors, which remain to be identified.

### Mediator and the expression of cell wall adhesins in different yeast species

Med31, Med20 and Srb9/Med13 all positively regulate the expression of the *ALS1* and *ALS3* cell wall adhesins in *C. albicans*, and Srb9/Med13 is further required for wild type expression of *HWP1*. Consistent with the transcriptional defects, the mutants are defective for biofilm formation, an adhesin-dependent phenotype [65], . Moreover, our genetic data supports the notion that the regulation of *ALS1* by Med31 is biologically relevant for biofilm development. The activators that mediate the effects of Mediator on adhesin transcription remain to be characterised. The results that Med31 is required for Ace2-dependent transcription, and that Ace2 has a role in *ALS3* expression suggest a potential role for Ace2 in Med31-dependent regulation of *ALS3* (Figure 2). The s*rb9/med13ΔΔ* mutant showed parallels in regards to both morphogenesis and gene expression phenotypes with the mutant inactivated in the activator Bcr1. Both mutants are required for the expression of *ALS1*, *ALS3* and *HWP1*, and, as was seen for the *bcr1* mutant, the *srb9/med13ΔΔ* mutant is proficient for hyphal growth in liquid media, but shows biofilm defects (Figure 6 and [65], [66]).

The biochemical, genetic and gene expression data in yeast supports the notion that Mediator is universally required for RNA polymerase II-dependent transcription (reviewed in [12]), and we therefore suggest that Mediator is directly involved in the transcription of the adhesin genes. Chromatin immunoprecipitation (ChIP) experiments to address occupancy of the *ALS* gene promoters by Med31 remained inconclusive, as we observed variability between biological replicates, from minimal to large 20–30 fold enrichments (data not shown). We suspect that this variability could be due to the exact timing of the crosslinking of Med31 to the promoters in respect to the transcriptional activation of the *ALS* genes in hyphae and/or how uniformly filamentous growth/*ALS* gene transcription is induced in the population of cells in the culture. ChIP studies in *S. cerevisiae* have yielded different results between labs in regards to Mediator occupancy, from modest (albeit functionally important) enrichments at some constitutively transcribed genes [75], no enrichment of Mediator subunits on the majority of transcribed genes [76], [77], to detectable enrichment of Mediator subunits upstream of many active, as well as inactive genes, and even in the coding regions of some genes [78]. More prominent enrichment for Mediator subunits is seen on genes that are responsive to stress (*e.g.* heat shock, or change of carbon source from glucose to galactose) [75]–[77]. Mediator does not bind directly to DNA, which is likely to be a factor in ChIP experiments. It has also been proposed that the interactions of Mediator with promoters could be transient [77]. Moreover, different Mediator subunits can yield different fold enrichments over the background (for example see [76], [77]), and it is therefore possible that a Mediator subunit other than Med31 needs to be assayed to detect consistent Mediator occupancy on promoters in *C. albicans*. In addition to a direct role for Mediator in adhesin gene expression, an alternative (and not mutually exclusive) possibility is that Mediator regulates the expression of transcription factors, which then in turn regulate the adhesins. The expression of several transcription factors was lower in *med31ΔΔ* mutants (Table 1, Dataset S2), for example that of *EFG1*, *TEC1* and *CPH1*, which have been previously shown to regulate the expression of Med31-regulated adhesins *ALS1*, *ALS3* and *EAP1* [59], [79].

In *S. cerevisiae* Med31 is a repressor of the adhesin *FLO11* (Figure 8), suggesting different functions for this Mediator subunit in the expression of cell wall adhesion molecules and regulation of adhesion-dependent phenotypes in comparison to *C. albicans*. In the *S. cerevisiae med31Δ* mutant, the reminder of the Mediator complex is intact [43], supportive of a specific role for Med31 in the repression of the *FLO11* gene that does not result simply from the loss of other repressor subunits from the Mediator complex. Previous reports in *S. cerevisiae* for Srb9/Med13 also show repression of the *FLO* adhesins [25], which is again opposite to the activating function for Srb9/Med13 in the expression of *ALS1/3* and *HWP1* that we observed in *C. albicans*. Other subunits of the Mediator Kinase domain as well as Sin4/Med16 from the Tail also inhibit expression of the *S. cerevisiae FLO* genes [25], [40]. In *Schizo. pombe*, the Mediator Kinase domain subunits Srb10 and Srb8/Med12 also repress the cell wall adhesins, despite the fact that some of the affected adhesins are not related to the *FLO* genes [28].

While the *FLO* and *ALS* gene are not related, there is conservation in terms of the pathways and transcription factors that regulate their expression in *S. cerevisiae* and *C. albicans* respectively. Examples include positive regulation by activators Flo8, Ste12/Cph1 and Tec1, and negative regulation by repressors Nrg1, Tup1 and Sfl1 [50], [59], [80]–[83]. Mediator interacts with gene-specific transcription factors to regulate gene expression [76], [84]. It is possible that the key transcription factor(s) through which Med31 and Srb9/Med13 act to regulate the adhesin-encoding genes differ between the two yeasts. How Mediator subunits repress the *S. cerevisiae FLO* genes is not well understood, but a suggested mechanism involves functional interactions with the repressors Sfl1 and Tup1 [85]. Both Sfl1 and Tup1 are required for repression of the *ALS* genes in *C. albicans* [50], [81], but the requirement for specific Mediator subunits in the repression of adhesins by these factors in *C. albicans* could differ from what has been suggested in *S. cerevisiae*. Another possibility relates to silencing mechanisms. The *FLO* genes in *S. cerevisiae* are subject to position-dependent silencing regulated by histone deacetylases ([86]; reviewed in [87]). The *C. albicans* adhesins are not known to be regulated by silencing. Mediator has been implicated in the regulation of chromatin structure and gene expression in regions affected by silencing, such as the sub-telomeres [12], [21]–[24]. Based on work in *S. cerevisiae*, it has been proposed that Mediator contributes to the establishment of a repressive chromatin structure by binding to the silenced regions and influencing the recruitment of the histone deaceytlase Sir2 and the histone acetyltransferase Sas2 and thus the acetylation status of histone H4 K16, a mark of active chromatin [24]. While this has not been tested directly, an interesting possibility is that the regulation of chromatin contributes to the repressive role of the Mediator subunits in the expression of the *S. cerevisiae FLO* genes. For example, the Tail subunit Sin4/Med16, which is a repressor of *FLO11* and of the sub-telomeric *FLO1* adhesin [40], [85], contributes to sub-telomeric silencing [24]. Med7 has also been found to affect chromatin structure, by regulating H4K16 acetylation and the presence of Sir2 and Sas2 at the sub-telomeres [24]. Med7 is closely functionally linked with Med31 - the N-terminal part of Med7 together with Med31 forms a structural and functional sub-module of Mediator, Med7N/31 [43]. It will be interesting to study whether differences in silencing mechanisms that operate on the *FLO* and *ALS* genes contribute to determining how Mediator subunits control their expression.

## Materials and Methods

### Yeast strains and growth conditions

The *C. albicans* strains used in this study are derivatives of BWP17 [88]. The *med31ΔΔ*, *srb9ΔΔ* and *med20ΔΔ* strains were constructed by standard methods based on PCR and homologous recombination, using *ARG4* and *URA3* as selective markers. The complemented strains were constructed by re-introducing a wild type copy of *MED31*, *SRB9* or *MED20* under own promoter and terminator into the *HIS1* locus of the respective mutants using the integrative plasmid pDDB78. To make matched *HIS1^+^* mutant strains, an empty pDDB78 vector was integrated into the genome of the respective strains. The *ace2* mutant is a homozygous mutant in the BWP17 strain background and was a generous gift from Aaron Mitchell (this strain is also *URA3^+^ ARG4^+^ HIS1^+^*). The *med31ΔΔ* +*TEF1*-*ALS1* overexpression strain was constructed using the plasmid pCJN498, as described in [65]. The *S. cerevisiae med31Δ* mutant was constructed in the ∑1278b strain using the KANMX4 cassette. The wt and *flo11Δ* mutant of Σ1278b were a generous gift from Todd Reynolds and are described in [67].

Standard growth conditions were YPD (2% glucose, 2% peptone, 1% yeast extract), at 30°C, 200 rpm. For *ura^−^* strains the media was supplemented with 80 µg/ml uridine. The mutants were selected using minimal media lacking the appropriate amino acids. The *TEF1-ALS1* overexpression strains have a nourseothricin resistance cassette (NAT) and were selected on 400 µg/ml NAT plates (nourseothricin was from Werner Bioagents). The *S. cerevisiae* mutant was selected on 200 µg/ml G418 plates.

For cell morphology analysis (Figure 3A), cells were classified as being in a chain if 3 or more cells were attached. An average of 200 cells per sample were counted, and the experiments were repeated at least with 3 independent cultures. The average and the standard error are shown in the figure. To observe the mother-bud junctions, cells were stained with calcofluor white (1 mg/ml) for 8 min in the dark, followed by washes in phosphate buffered saline (PBS). Imaging was done using an Olympus IX81 microscope with the Olympus cell∧M software, using the 100× objective with DIC or the DAPI filter for calcofluor white stained cells.

Filamentous growth was tested by dilution of cells from overnight cultures grown to OD^600^ = 0.1–0.2 into pre-warmed YPD+10% calf serum, Spider media (1% nutrient broth, 1% D-mannitol, 2 g K_2_HPO_4_), M199 or N-acetylglucosamine media (9 g NaCl, 6.7 g yeast nitrogen base and 0.56 g N-acetylglucosamine per liter) and incubated at 37°C for the times indicates in the figures. All cell imaging was done using an Olympus IX81 microscope with the Olympus cell∧M software. For testing filamentation on plates, *C. albicans* strains were re-streaked on plates containing filamentous-growth inducing media. Plates were incubated for up to five days at 37°C and colonies examined and photographed with a stereo dissecting microscope (Olympus SZX 16).

For analysis of sensitivities to various drugs and chemicals, ten fold serial dilutions of cultures from wild type and mutant strains were dropped on control plates, or plates containing the compounds indicated in Figure S4 and Table 3. Plates were incubated at 30°C for three days (unless growth was assessed at 37°C or 16°C), and photographed.

### Biofilm assays and imaging

*C. albicans* biofilms were grown in 96-well microtiter plates or on silicone disks for quantitative or qualitative analysis respectively. Quantitative biofilm assays were performed as described [89], [90]. 100 ml of cultures of *C. albicans* wild type or mutant strains (10^7^ cells/ml in Spider media) were added to wells and incubated at 37°C with gentle shaking (75 rpm) for 90 min (adhesion phase). Non-adherent cells were discarded and 100 µl of fresh Spider media were added to each of the wells. Biofilms were allowed to develop for a future 4.5 h (6 h in total), 24 h, and 48 h, representing the early, intermediate or mature stage of biofilm development respectively. The medium was replenished after 24 h by aspiration and addition of fresh medium. Biofilm biomass was determined at the different time points using crystal violet staining. Wells containing only Spider medium with no yeast served as negative controls. For qualitative studies, biofilms were formed *in vitro* on serum-treated silicone disks, which is a well-established system for biofilm analysis [66]. Sterile silicone disks were pretreated with fetal bovine serum (Sigma) overnight at 37°C with gentle shaking (75 rpm). The silicone disks were then washed twice with PBS and transferred to a 12-well plate containing 2 ml of freshly prepared cell suspensions (10^7^ cells/ml in Spider media). The plate was incubated for 1.5 h at 37°C with gentle shaking (75 rpm) to allow the yeast to adhere to the disk surfaces. The silicone disks were then washed in PBS and transferred to a new 12-well plate with Spider media followed by incubation for 48 h at 37°C with shaking at 75 rpm. The established biofilms were examined with SEM or CLSM. For SEM, biofilms were fixed with glutaraldehyde (2.5%, v/v, in 0.1 M cacodylate buffer, pH 7.0) and 1% osmium tetraoxide at room temperature, and dehydrated with gradually increased ethanol (50%, 75%, 95%, 100%, and absolute 100%) and hexamethyldisilazane (HMDS) (50%, 75%, 95%, 100%, and absolute 100%). Samples were coated with gold with a Balzers SCD005 sputter coater and viewed under a Hitachi S570 scanning electron microscope. For CSLM, biofilms were stained with FUN-1 (10 µM, Molecular Probes) and Concanavalin A–Alexafluor488 conjugate (Con A, 25 µg ml^−1^; Molecular Probes) for 45 min at 37°C. Stained biofilms were observed with a Leica SP5 CLSM, and images were captured and processed using the softwares Leica LAS AF and Amira 5.2.1. All experiments for quantitative analysis were repeated at least three times in triplicate, and qualitative assays were repeated at least 3 times. One-way ANOVA was used to compare the difference in biofilm biomass produced by different *C. albicans* strains. p values of <0.05 were considered to be statistically significant.

For *S. cerevisiae* biofilms (Figure 8), overnight cultures were growth in synthetic complete media supplemented with 2% glucose. The cells were then resuspended into 96 well polystyrene plates to an OD^600^ = 1.0 using synthetic 0.2% glucose media [67]. Adherence was assayed at the time points indicated in the figure using crystal violet staining as described above.

### RNA extraction, quantitative PCR, and microarray analysis

RNA was extracted using the hot-phenol method from cultures grown in YPD at 30°C to log phase (OD^600^ = 1). Following hot-phenol extraction, 100 µg of RNA samples were further purified using the RNAeasy kit and following the manufacturer's instructions. The microarray analysis was performed as described [91], on microarrays spotted with 6459 70-mer oligonucleotides (GEO Platform GPL9818). Data normalization and analysis was conducted in GeneSpring GX version 7.3 (Agilent Technologies). The microarray data set has been deposited in GEO, under accession number GSE31632. Genes that were up- or down-regulated in the *med31ΔΔ* mutant by 1.5 fold or more (p≤0.05) were selected from a Volcano Plot and considered to be differentially expressed. Gene ontology analysis was performed at the Candida genome database (CGD, candidagenome.org) [92]. GSEA analysis ([93] and Sellam et al, submitted) was performed using the GseaPreranked tool and the weighted enrichment statistics on 6387 (for *C. albicans*) gene sets each containing 5–500 genes. Statistical significance was estimated from 1000 permutations. Enrichment maps were constructed with Cytoscape 2.8 (http://www.cytoscape.org; [94]) and the Enrichment Map 1.1 plug-in (http://baderlab.org/Software/EnrichmentMap) using the default settings. For quantitative PCR, reverse transcription was performed using the Transcriptor High Fidelity cDNA synthesis kit from Roche. qPCR reactions were prepared using Fast-Start Sybr Green Master (Roche) on an Eppendorf Realplex master cycler and analysed by absolute quantification. The expression levels of the mRNAs were normalized to the level of *ACT1* or the GAPDH encoding gene *TDH3*. Three independent cultures were analyzed, with two technical replicates each. qPCR primers that enable differential amplification of the *ALS1* and *ALS3* genes were from Green et al [95]. Sequences for all qPCR primers used in this study are listed in Table S2. For analysis of gene expression in YPD, cultures were grown under the same condition as for the microarrays analysis, to mid log phase (OD^600^ = 1). For assaying expression of genes under hyphal growth, strains were grown in Spider media as described in [65].

### Worm infection assays

The worm-*C. albicans* infection assay was performed as described previously [64]. Briefly, young adult nematodes were allowed to feed for 4 h on lawns of *C. albicans* grown on solid BHI media (Difco) containing ampicillin (100 µg/ml), kanamycin (50 µg/ml) and streptomycin (200 µg/ml). Worms were washed with M9 media and transferred into wells of a six-well microtiter dish (Corning) containing 2 ml of liquid media (80% M9 and 20% BHI) at 60 to 80 worms per well. The plates were incubated at 25°C, and worms were qualitatively assessed at 24 h intervals for penetrative *C. albicans* filamentation using a DIC microscope and photographed using an Olympus IX81 microscope with the Olympus cell∧M software. The % of worms with penetrative filamentation was determined from four independent experiments at day 3 of the infection. Means and the standard error were calculated, and the p value was determined using the student *t*-test. The killing assays (Figure 4C) were performed three times and equivalent results were obtained.

## Supporting Information

Dataset S1 The complete transcriptome analysis data for the *med31ΔΔ* mutant.
(XLS)

Dataset S2 Gene ontology and other functional analysis of the *med31ΔΔ* mutant transcriptome.
(XLSX)

Figure S1 Analysis of the *med31ΔΔ* transcriptome data A) Chromosomal view of the transcriptional profile of the *med31ΔΔ* mutant of *C. albicans*. B) Scatter plot comparisons of the *med31ΔΔ* transcriptome to changes in gene expression observed upon oxidative or osmotic stress in *C. albicans*.
(TIF)

Figure S2 A complete GSEA network of genes differentially expressed in the *med31ΔΔ* mutant of *C. albicans*. The gene categories can be viewed by zooming in.
(PDF)

Figure S3 qPCR analysis of gene expression in Mediator mutants of *C. albicans*. Cells were grown in YPD at 30°C for yeast growth or Spider at 37°C for hyphal growth and gene expression analysed as described in the Materials and Methods. The levels of the indicated genes were normalised to the levels of the glyceraldehyde phosphate dehydrogenase (GAPDH)-encoding gene *TDH3*. Shown are averages of three independent experiments and the standard error.
(TIF)

Figure S4 Sensitivities of the *C. albicans* Mediator mutants to various stresses. 10 fold serial dilutions of the wild type, Mediator mutants and complemented strains were dropped on YPD plates containing the indicated compounds. The plates were incubated at 30°C (unless stated otherwise) for 3–4 days and photographed. The mutants were scored as sensitive or resistant and the results are presented in Table 3.
(TIF)

Figure S5 Filamentation defect of the *med31ΔΔ* mutant in liquid media. Overnight cultures of the indicated strains were diluted into media pre-warmed at 37°C and the appearance of filamentous cells was monitored over time. The images were taken with a 100× magnification objective and the scale bar represents 10 µm. In Spider media, the mutant has a pronounced filamentation defect, while in the other media filamentation by the mutant is somewhat delayed, and a larger number of cell chains and pseudohyphae is observed than in the complemented strain.
(TIF)

Figure S6 The *med31ΔΔ* mutant has a filamentation defect in the worm infection assay even after prolonged incubation. Worms infected with the wild type, the *med31ΔΔ* mutant or the complemented *med31ΔΔ+MED31* strain were imaged 7 days post infection using an Olympus IX81 microscope.
(TIF)

Figure S7 Scanning electron microscopy of wild type and *med31ΔΔ* mutant biofilms. Biofilms were formed *in vitro* on serum coated silicone disks. Mature biofilms (48 h) were imaged by scanning electron microscopy (SEM) as described in Materials and Methods. The SEM experiments confirmed the biofilm defect of the *med31ΔΔ* mutant observed by confocal microscopy (Figure 5).
(TIF)

Figure S8 Ectopic expression of *ALS1* does not complement the growth defect of the *med31ΔΔ* mutant. Cultures from the indicated strains were grown in either rich YPD (upper panel) or minimal synthetic complete media (lower panel). Growth was assessed by measuring OD^600^ at regular intervals over an 8 h time course. Three independent clones of the *med31ΔΔ+TEF1-ALS1* strain were tested, all of which rescued the biofilm formation defect of the *med31ΔΔ* mutant, but not the growth defect.
(TIF)

Table S1 Comparison of the transcriptome changes in the *C. albicans med31ΔΔ* mutant and those observed upon inactivation of Ace2. The *ace2* data is from [57].
(DOC)

Table S2 The list of primers used in this study.
(DOC)
