# Supplementary figures and images for: The Functions of Mediator in Candida albicans Support a Role in Shaping Species-Specific Gene Expression
Source: PLoS Genet. 2012 Apr 5;8(4):e1002613. doi: 10.1371/journal.pgen.1002613 (PMC3320594; doi:10.1371/journal.pgen.1002613)

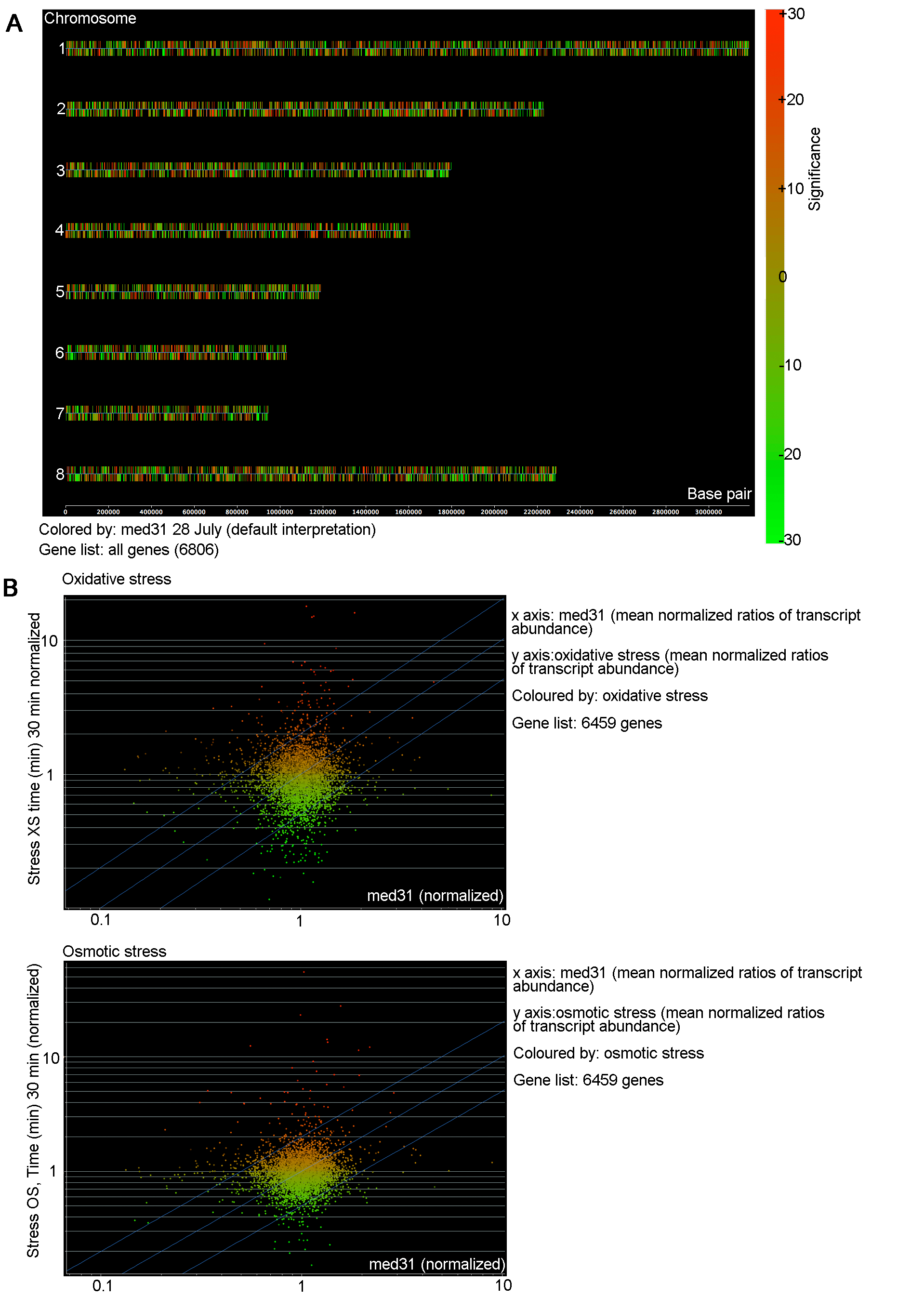

Supplement: Figure S1 — Analysis of the med31ΔΔ transcriptome data A) Chromosomal view of the transcriptional profile of the med31ΔΔ mutant of C. albicans. B) Scatter plot comparisons of the med31ΔΔ transcriptome to changes in gene expression observed upon oxidative or osmotic stress in C. albicans. (TIF) [file pgen.1002613.s003.tif]

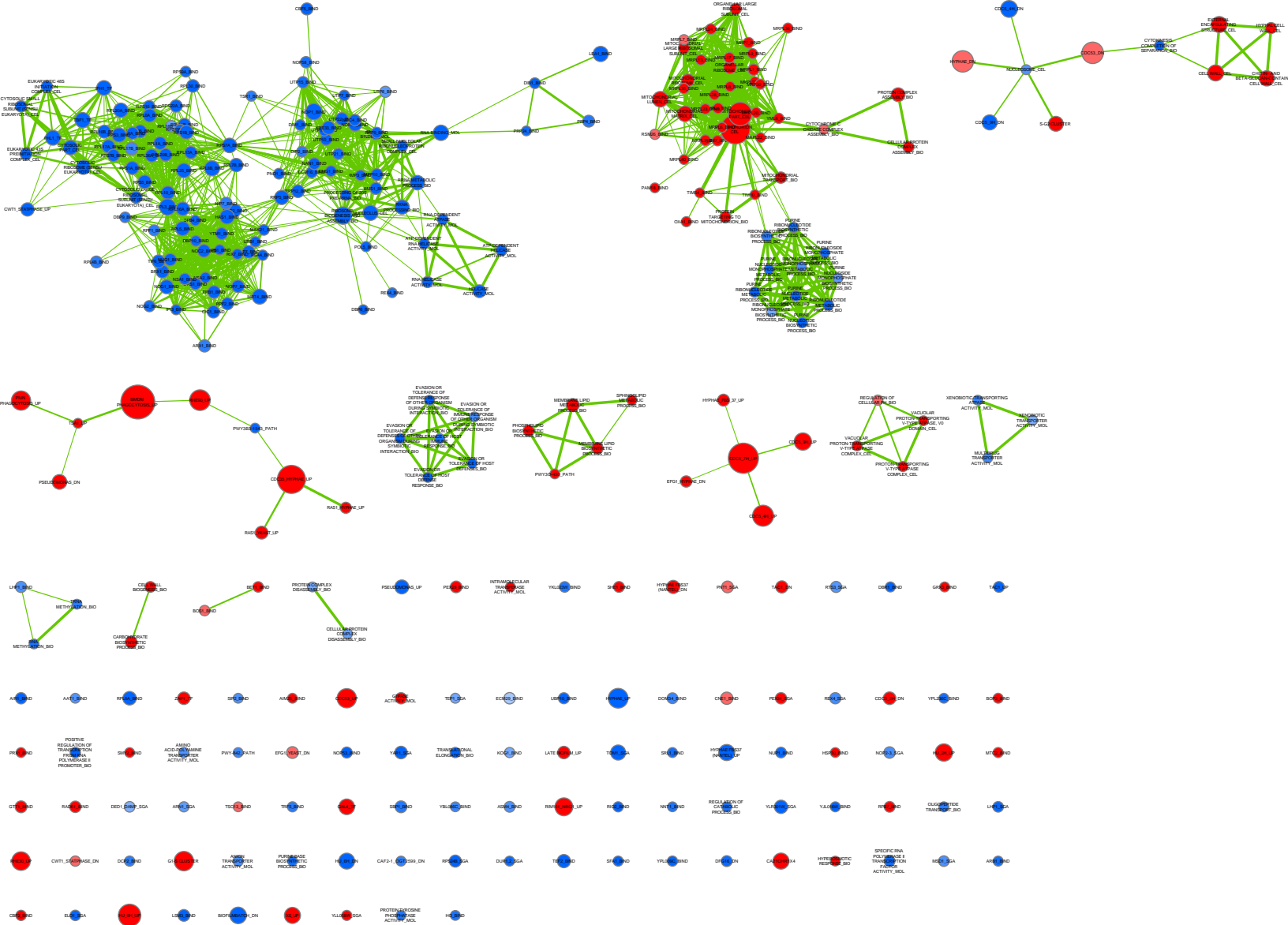

Supplement: Figure S2 — A complete GSEA network of genes differentially expressed in the med31ΔΔ mutant of C. albicans. The gene categories can be viewed by zooming in. (PDF) [file pgen.1002613.s004.pdf]

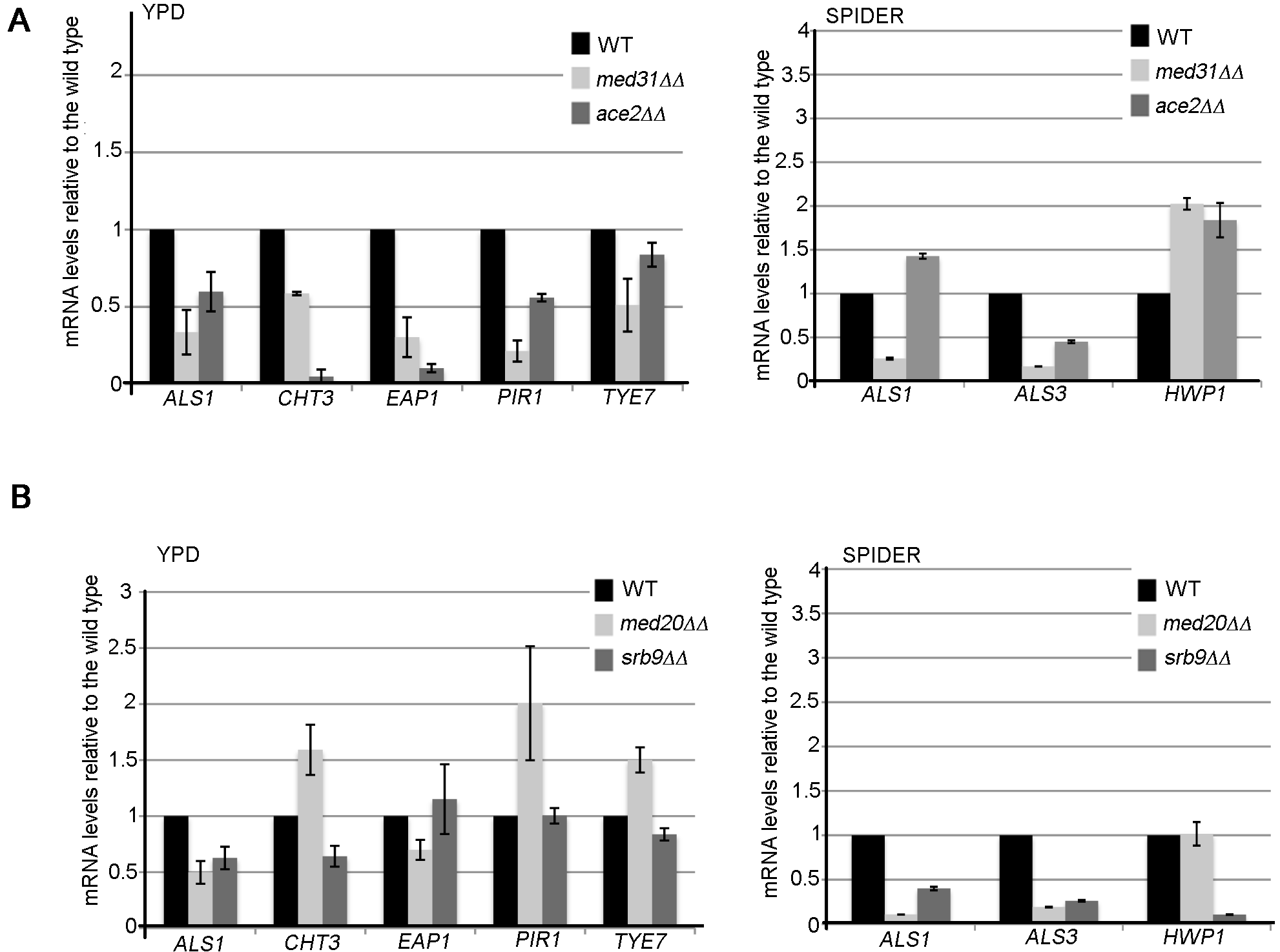

Supplement: Figure S3 — qPCR analysis of gene expression in Mediator mutants of C. albicans. Cells were grown in YPD at 30°C for yeast growth or Spider at 37°C for hyphal growth and gene expression analysed as described in the Materials and Methods. The levels of the indicated genes were normalised to the levels of the glyceraldehyde phosphate dehydrogenase (GAPDH)-encoding gene TDH3. Shown are averages of three independent experiments and the standard error. (TIF) [file pgen.1002613.s005.tif]

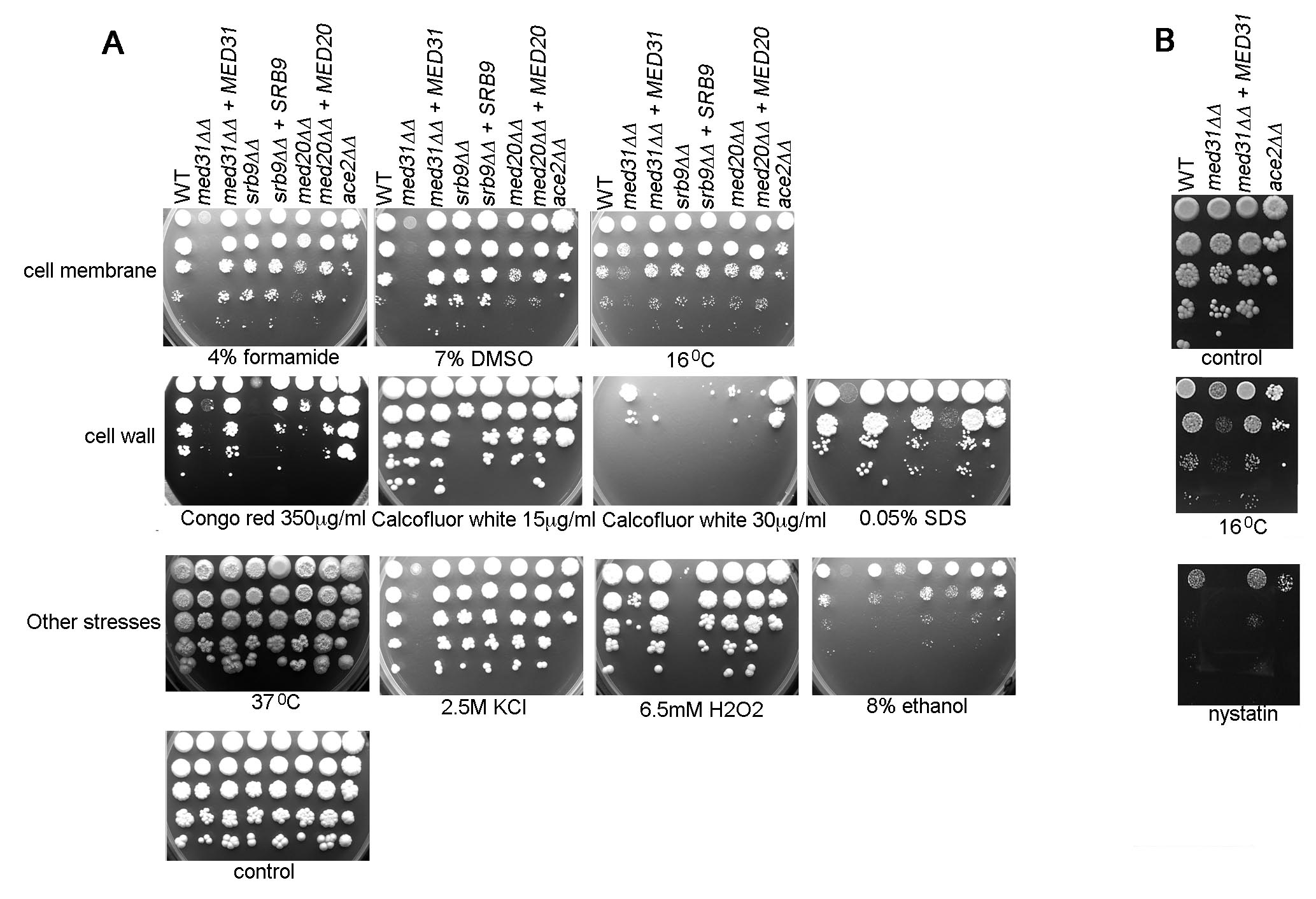

Supplement: Figure S4 — Sensitivities of the C. albicans Mediator mutants to various stresses. 10 fold serial dilutions of the wild type, Mediator mutants and complemented strains were dropped on YPD plates containing the indicated compounds. The plates were incubated at 30°C (unless stated otherwise) for 3–4 days and photographed. The mutants were scored as sensitive or resistant and the results are presented in Table 3. (TIF) [file pgen.1002613.s006.tif]

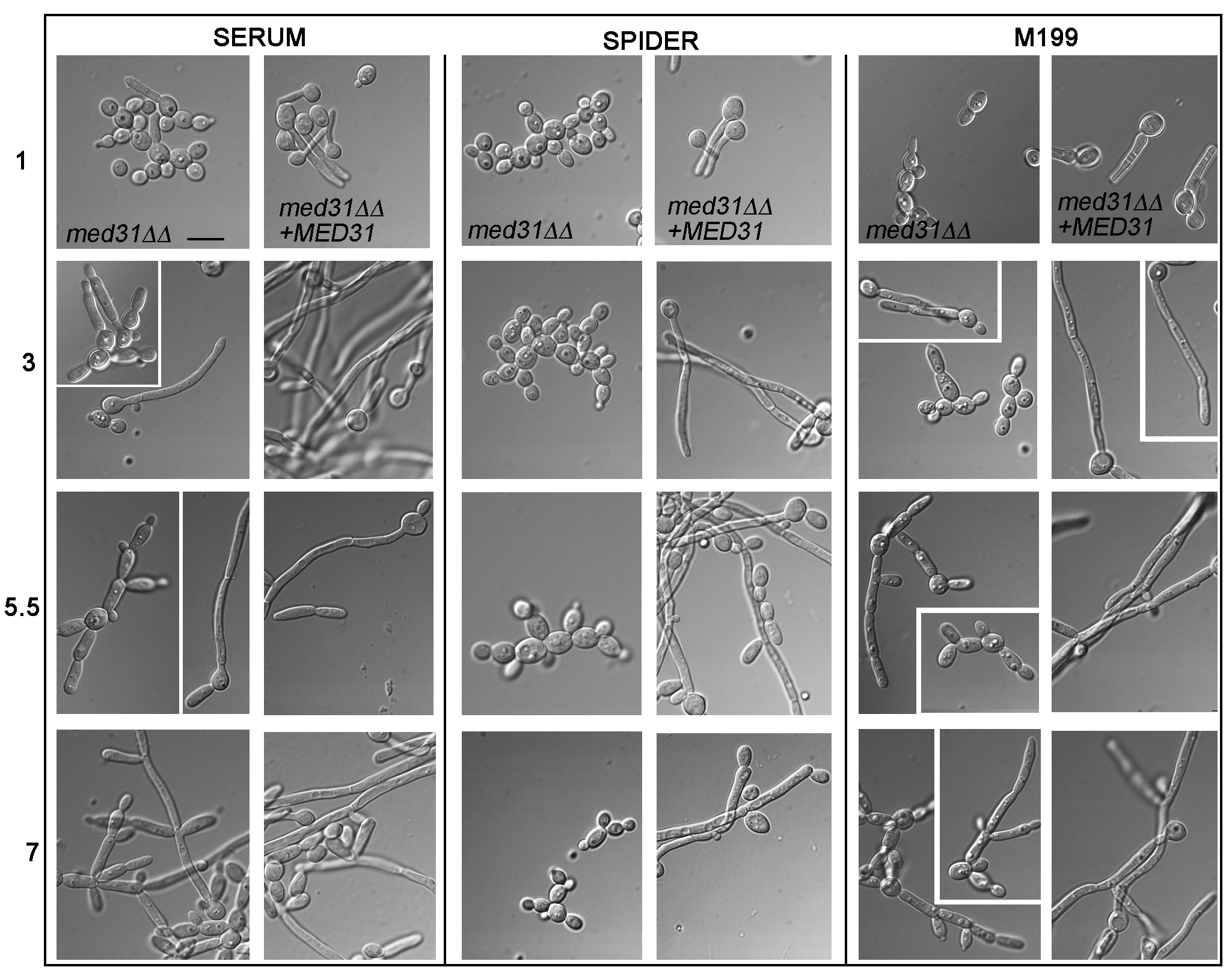

Supplement: Figure S5 — Filamentation defect of the med31ΔΔ mutant in liquid media. Overnight cultures of the indicated strains were diluted into media pre-warmed at 37°C and the appearance of filamentous cells was monitored over time. The images were taken with a 100× magnification objective and the scale bar represents 10 µm. In Spider media, the mutant has a pronounced filamentation defect, while in the other media filamentation by the mutant is somewhat delayed, and a larger number of cell chains and pseudohyphae is observed than in the complemented strain. (TIF) [file pgen.1002613.s007.tif]

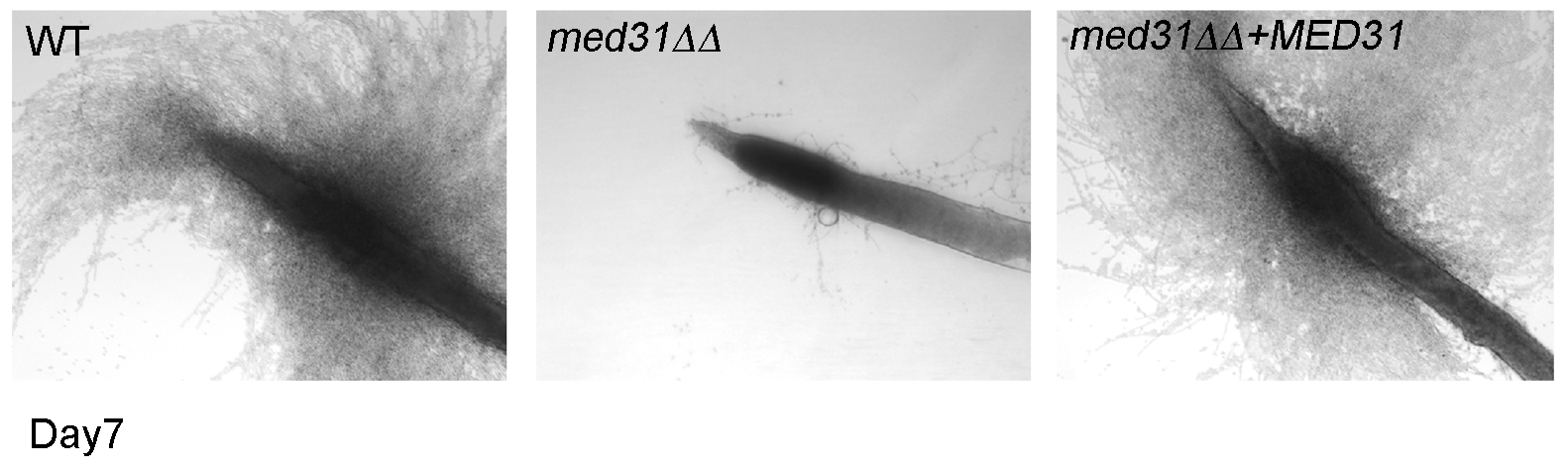

Supplement: Figure S6 — The med31ΔΔ mutant has a filamentation defect in the worm infection assay even after prolonged incubation. Worms infected with the wild type, the med31ΔΔ mutant or the complemented med31ΔΔ+MED31 strain were imaged 7 days post infection using an Olympus IX81 microscope. (TIF) [file pgen.1002613.s008.tif]

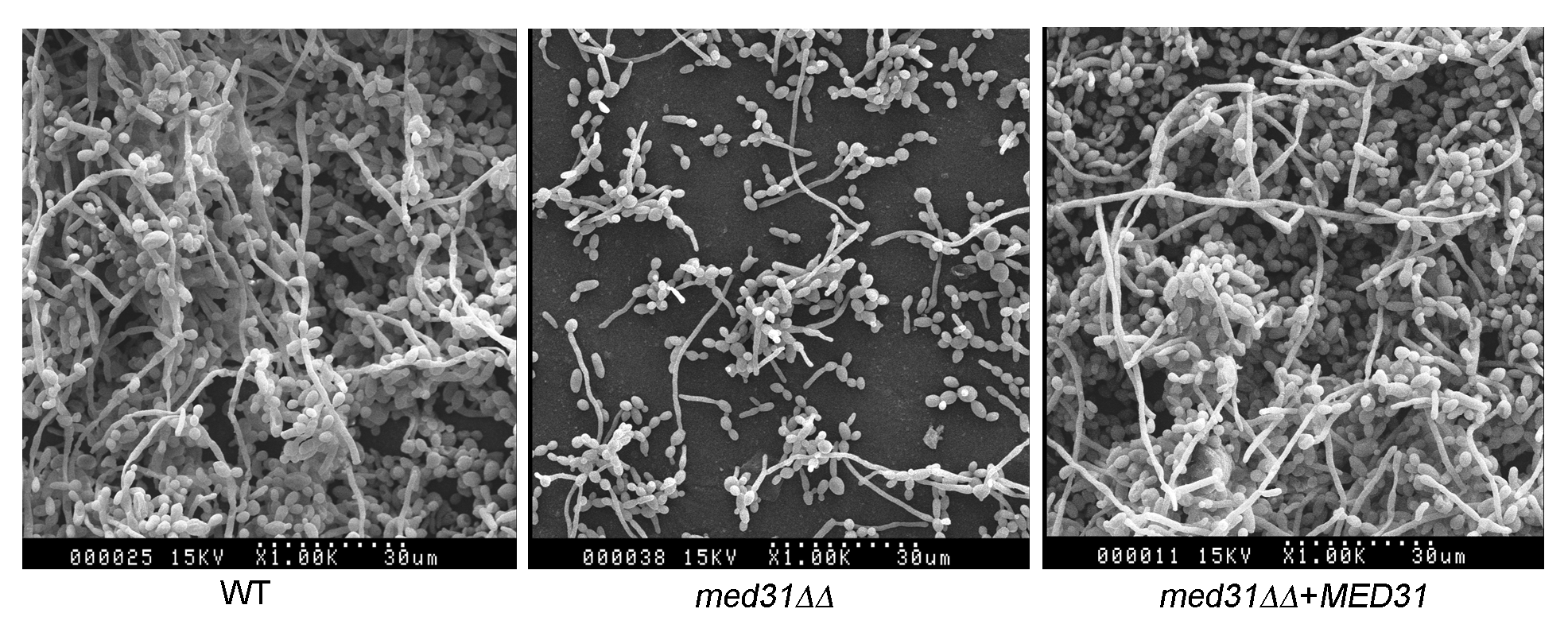

Supplement: Figure S7 — Scanning electron microscopy of wild type and med31ΔΔ mutant biofilms. Biofilms were formed in vitro on serum coated silicone disks. Mature biofilms (48 h) were imaged by scanning electron microscopy (SEM) as described in Materials and Methods. The SEM experiments confirmed the biofilm defect of the med31ΔΔ mutant observed by confocal microscopy (Figure 5). (TIF) [file pgen.1002613.s009.tif]

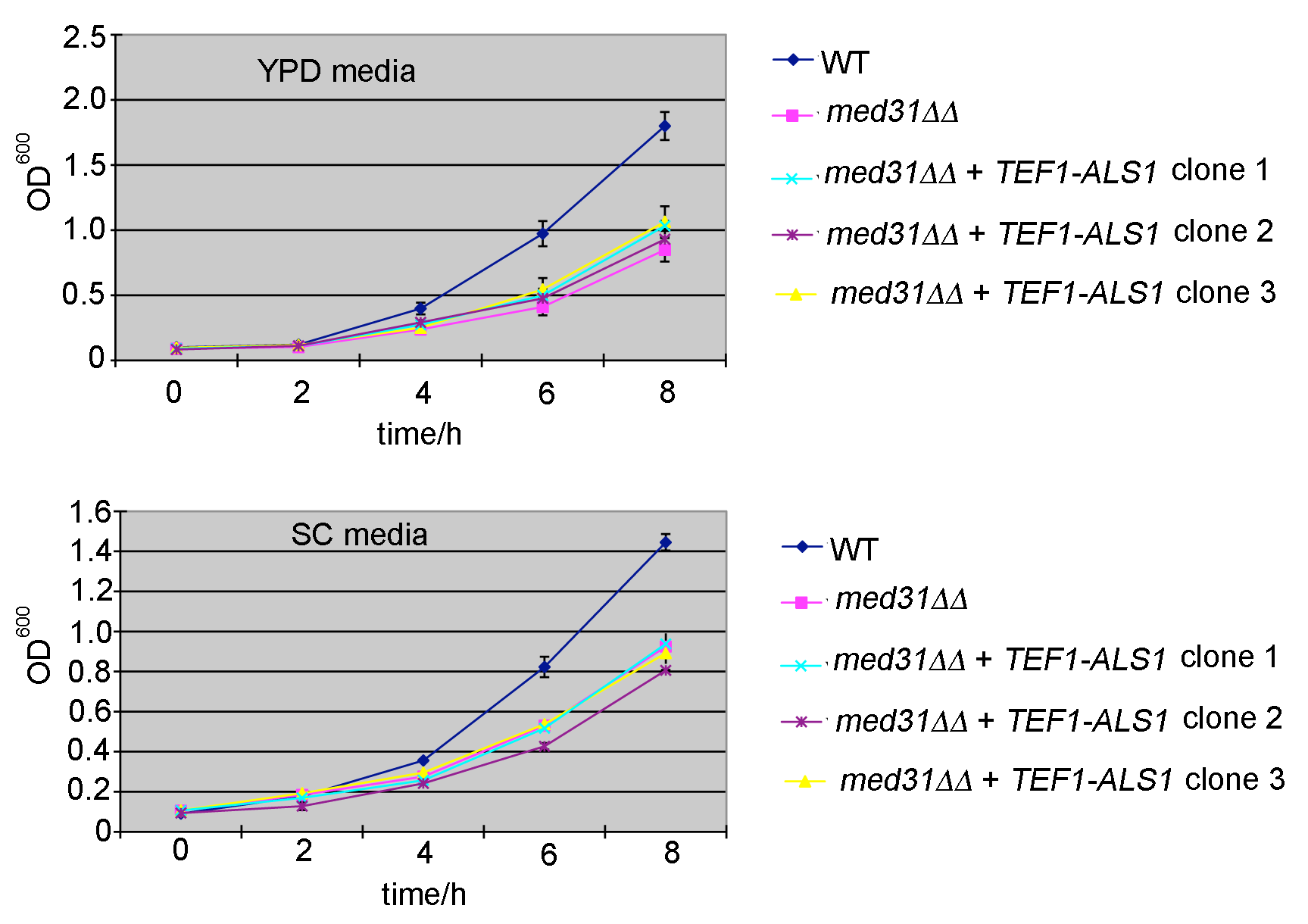

Supplement: Figure S8 — Ectopic expression of ALS1 does not complement the growth defect of the med31ΔΔ mutant. Cultures from the indicated strains were grown in either rich YPD (upper panel) or minimal synthetic complete media (lower panel). Growth was assessed by measuring OD600 at regular intervals over an 8 h time course. Three independent clones of the med31ΔΔ+TEF1-ALS1 strain were tested, all of which rescued the biofilm formation defect of the med31ΔΔ mutant, but not the growth defect. (TIF) [file pgen.1002613.s010.tif]
