# Supplementary material for: The Functions of Mediator in Candida albicans Support a Role in Shaping Species-Specific Gene Expression
Source: PLoS Genet. 2012 Apr 5;8(4):e1002613. doi: 10.1371/journal.pgen.1002613 (PMC3320594; doi:10.1371/journal.pgen.1002613)
Supplement: Table S1 — Comparison of the transcriptome changes in the C. albicans med31ΔΔ mutant and those observed upon inactivation of Ace2. The ace2 data is from [57]. (DOC) [file pgen.1002613.s011.doc]

| **ORF** | **Gene name** | **Description/Function** | **Change in expression** | |
| --- | --- | --- | --- | --- |
|  |  |  | *med3111* | *ace2* |
| **Up-regulated** | | | | |
| *orf19.4255* | *ECM331* | Involved in cell wall biogenesis and architecture | 2.64 | 1.84 |
| *orf19.4082* | *DDR48* | Immunogenic stress-associated protein | 2.18 | 5.21 |
| *orf19.1300* |  | Putative protein of unknown function | 2.06 | 1.54 |
| *orf19.3797* |  | Structural constituent of mitochondrial ribosome | 1.94 | 3.33 |
| *orf19.1485* |  | Structural constituent of mitochondrial ribosome | 1.72 | 2.38 |
| *orf19.4751* |  | Structural constituent of mitochondrial ribosome | 1.67 | 2.99 |
| *orf19.5235* |  | Putative protein of unknown function | 1.67 | 3.8 |
| *orf19.2887* |  | Putative protein of unknown function | 1.64 | 2.28 |
| *orf19.3480* |  | Structural constituent of mitochondrial ribosome | 1.59 | 2.55 |
| *orf19.7012* |  | Structural constituent of mitochondrial ribosome | 1.59 | 3.03 |
| *orf19.4204* |  | Structural constituent of mitochondrial ribosome | 1.59 | 2.3 |
| *orf19.755* | *MRPL37* | Structural constituent of mitochondrial ribosome | 1.57 | 2.39 |
| *orf19.1195* |  | Mitochondrial peptidase Oct1 | 1.55 | 1.71 |
| *orf19.185* |  | Structural constituent of ribosome, mitochondrial translational initiation | 1.54 | 2.97 |
| *orf19.439* |  | Similar to Mhr1, mtDNA recombination | 1.54 | 1.92 |
| **Down-regulated** | | | | |
| *orf19.5610* | *ARG3* | [Ornithine carbamoyltransferase activity](http://www.candidagenome.org/cgi-bin/GO/go.pl?goid=4585) | -1.51 | -1.71 |
| *orf19.5917* | *STP3* | Transcription factor | -1.57 | -1.9 |
| *orf19.1277* |  | Putative protein of unknown function | -1.58 | -1.67 |
| *orf19.2758* | *PGA38* | Putative GPI anchored protein, adhesin-like | -1.6 | -10.21 |
| *orf19.5615* | *AYR2* | Putative protein of unknown function | -1.64 | -1.7 |
| *orf19.3664* | *HSP31* | Putative protein of unknown function | -1.66 | -1.44 |
| *orf19.3066* | *ENG1* | [Glucan endo-1,3-beta-D-glucosidase activity](http://www.candidagenome.org/cgi-bin/GO/go.pl?goid=42973) | -1.75 | -2.8 |
| *orf19.1206* | *FET35* | Putative multicopper ferroxidase | -1.84 | -2.63 |
| *orf19.3749* | *IFC3* | [Oligopeptide transporter activity](http://www.candidagenome.org/cgi-bin/GO/go.pl?goid=15198) | -2.03 | -2.27 |
| *orf19.54* | *RHD1* | Putative beta-mannosyltransferase | -2.14 | -1.51 |
| *orf19.220* | *PIR1* | [Structural constituent of cell wall](http://www.candidagenome.org/cgi-bin/GO/go.pl?goid=5199) | -2.16 | -4.25 |
| *orf19.7586* | *CHT3* | [Chitinase](http://www.candidagenome.org/cgi-bin/GO/go.pl?goid=4568) | -2.41 | -3.45 |
| *orf19.6720* |  | Putative protein of unknown function | -2.66 | -1.48 |
| *orf19.4438* | *RME1* | Similar to *S. cerevisiae* Rme1 | -2.73 | -2.26 |
| *orf19.7085* |  | Putative protein of unknown function | -2.73 | -1.79 |
| *orf19.3803* | *MNN22* | Golgi alpha-1,2-mannosyltransferase | -3.21 | -2.43 |
| *orf19.3893* | *SCW11* | Cell wall protein | -3.54 | -10.61 |
| *orf19.822* |  | Putative protein of unknown function / abnormal biofilm detachment in mutant | -4.06 | -2.08 |
| *orf19.5282* |  | Putative protein of unknown function | -4.52 | -1.55 |
| *orf19.1189* |  | Putative protein of unknown function | -5.67 | -1.68 |

**Table S1.**
