# Supplementary material for: The Functions of Mediator in Candida albicans Support a Role in Shaping Species-Specific Gene Expression
Source: PLoS Genet. 2012 Apr 5;8(4):e1002613. doi: 10.1371/journal.pgen.1002613 (PMC3320594; doi:10.1371/journal.pgen.1002613)
Supplement: Table S2 — The list of primers used in this study. (DOC) [file pgen.1002613.s012.doc]

| ***Saccharomyces cerevisiae* qPCR primers** | | | | |  |
| --- | --- | --- | --- | --- | --- |
| **Primer Name** | **Gene/ORF** | | | **Sequences 5' --> 3'** | **Reference** |
| ScACT1 Forward | ACT1 | | | CGTCTGGATTGGTGGTTCTA | This study |
| ScACT1 Reverse | ACT1 | | | GATGGACCACTTTCGTCGTA | This study |
| Flo11 Forward | FLO11 | | | AGA CTT CAA TTG GCA CAT GG | This study |
| Flo11 Reverse | FLO11 | | | GAA CCC AAG AAA CGG AAG TC | This study |
| ***Candida albicans* qPCR primers** | | | | | |
| **Primer Name** | | **Gene/ORF** | **Sequences 5' --> 3'** | | **Reference** |
| CaACT1 Forward | | ACT1/orf19.5007 | CCCAGGTATTGCTGAACGTA | | Dagley et al., 2011 |
| CaACT1 Reverse | | ACT1/orf19.5007 | GAACCACCAATCCAGACAGA | | Dagley et al., 2011 |
| ALS1 Forward | | ALS1/orf19.5741 | TTCTCATGAATCAGCATCCACAA | | Green et al., 2005 |
| ALS1 Reverse | | ALS1/orf19.5741 | CAGAATTTTCACCCATACTTGGTTTC | | Green et al., 2005 |
| ALS3 Forward | | ALS3/orf19.1816 | AATGGTCCTTATGAATCACCATCTACT | | Green et al., 2005 |
| ALS3 Reverse | | ALS3/orf19.1816 | GAATTTTCATCCATACTTGATTTCACA | | Green et al., 2005 |
| HWP1 Forward | | HWP1/orf19.1321 | AATCCTCCTCAACCTGATCAGCCTG | | This study |
| HWP1 Reverse | | HWP1/orf19.1321 | AGCTGGAGTTGTTGGCTTTTCTGGA | | This study |
| EAP1 Forward | | EAP1/orf19.1401 | TGCCCCAGGTACTGAAACCACTC | | This study |
| EAP1 Reverse | | EAP1/orf19.1401 | AGTGCCTGGGATAACGGGTTGAG | | This study |
| PIR1 Forward | | PIR1/orf19.220 | ACCAAACCGCCAAGGCCACT | | This study |
| PIR1 Reverse | | PIR1/orf19.220 | ACCGTCACTGATTTGAGCCACTGG | | This study |
| CHT3 Forward | | CHT3/orf19.7586 | AGGTGGTGCTGCTGGATCTTATGG | | This study |
| CHT3 Reverse | | CHT3/orf19.7586 | TGAGCAAATTGTTTGGCAGTGGCA | | This study |
| TYE7 Forward | | TYE7/orf19.4941 | AGAACCAGGTACGAAGGCAGCT | | This study |
| TYE7 Reverse | | TYE7/orf19.4941 | TGCCGGCAATCTTGGCATTAATGT | | This study |
| TDH3 Forward | | TDH3/orf19.6814 | TAAGAGTTGCTTTGGGCAGA | | This study |
| TDH3 Reverse | | TDH3/orf19.6814 | AATGACCAAGTCGTCACCAG | | This study |

| ***Candida albicans* deletion and over-expression primers** | | | | |
| --- | --- | --- | --- | --- |
| **Gene** | **ORF** | **Description** | **Sequences 5' --> 3'** | **Reference** |
| *MED31* | orf19.1249 | MED31 forward deletion primer | CAGAGAATATTTCCATGGCGAAAACAATTGAAAAAAAAAAGCACACACAACAAAGTTTCACCTAACCAACATCATTACTTTCAAACACTCACCATACCCTTTCCCAGTCACGACGTT | This study |
| *MED31* | orf19.1249 | MED31 reverse deletion primer | ATGTGTTTCCAGCAGTTAAATTAGTTACCAATGCAGCTACTTTTATTCTTGTCATTATTAACACTCTACTAGATCTTTTTGCTTCCTTCCTTTCATTTCTGTGGAATTGTGAGCGGATA | This study |
| *MED13/SRB9* | orf19.1451 | MED13 forward deletion primer | TTCAACTTCGAGGAATCTCATTTAACTTCAGCTTTCTTCTGGCTACTACAATCTTCACTTACAACTAGAATTCATACTAAAAACATTCGATAGAAACATCTTTCCCAGTCACGACGTT | This study |
| *MED13/SRB9* | orf19.1451 | MED13 reverse deletion primer | TGTAGCATTTGTTGAACAACGACTGGGAGGAAAAAAAAAAAATACAGAACTAAAAAAAGTCGTGCGAAATGGCCATACTCATTAAACCTATGTACAGCTTGTGGAATTGTGAGCGGAT | This study |
| *ALS1* | orf19.5741 | ALS1 forward ectopic expression primer | AATGAAAACGTTAAGACATTGGAATTTTTCATCAAATTTACGATGAATTGCTAATCATCTTTGGAGATATTCGTAGTAAGATCTTCAACCCAAGTTGTCAATCAAGCTTGCCTCGTCCCC | This study |
| *ALS1* | orf19.5741 | ALS1 reverse ectopic expression primer | TGGACCAAGTTAATGAATTAAAACTATCAAAAACACCAGTGATTGTCTTTGCACTTGCAATTGACAAATATAGGAATAACAATGTAAATTGTTGAAGCAT TATAAAATGTATACTTAGAA | This study |
| *MED20/SRB2* | orf19.2711.1 | MED20 forward deletion primer | GGTTTCTAGTTTAAGTAACAACATTGATGTTGAAAACAAGGGTGAATACTTCTTAACAGACCTCGCAACACTTTGTATATACTTTTCGAATCATTAGATTTTTCCCAGTCACGACGTT | This study |
| *MED20/SRB2* | orf19.2711.1 | MED20 reverse deletion primer | TGTCTTCTCCTTTAATGATAATATCTATGTGGTCAACTTACAATCTTTATTTCTTATATAATTTATTTTCATTTTCTCAAACTAATTACAGTTGTTGACGGTGGAATTGTGAGCGGATA | This study |

| ***Candida albicans* complementation primers** | | | | |
| --- | --- | --- | --- | --- |
| **Gene** | **ORF** | **Description** | **Sequences 5' --> 3'** | **Reference** |
| MED31 | orf19.1249 | MED31 forward complementation primer | TTCACACAGGAAACAGCTATGACCATGATTACGCCAAGCTAATTTAGCTTCAATTTCTTC | This study |
| MED31 | orf19.1249 | MED31 reverse complementation primer | TCGACCATATGGGAGAGCTCCCAACGCGTTGGATGCATAGTCCTCTATTATCAACATAAT | This study |
| MED13/SRB9 | orf19.1451 | MED13 forward complementation primer | TTCACACAGGAAACAGCTATGACCATGATTACGCCAAGCTAGTACATCTCTTTGCAAGTG | This study |
| MED13/SRB9 | orf19.1451 | MED13 reverse complementation primer | TCGACCATATGGGAGAGCTCCCAACGCGTTGGATGCATAGTGCAAAAGATGGTTCCTTTT | This study |
| MED20/SRB2 | orf19.2711.1 | MED20 forward complementation primer | TTCACACAGGAAACAGCTATGACCATGATTACGCCAAGCTTTTAAATTCAAAAGTGTAGA | This study |
| MED20/SRB2 | orf19.2711.1 | MED20 reverse complementation primer | TCGACCATATGGGAGAGCTCCCAACGCGTTGGATGCATAGAGGAAGACTATTCTAGAAAA | This study |

**Table S2.**
